# Uncharted digenean diversity in Lake Tanganyika: cryptogonimids (Digenea: Cryptogonimidae) infecting endemic lates perches (Actinopterygii: Latidae)

**DOI:** 10.1186/s13071-020-3913-x

**Published:** 2020-05-01

**Authors:** Nikol Kmentová, Rodney A. Bray, Stephan Koblmüller, Tom Artois, Els Lea R. De Keyzer, Milan Gelnar, Maarten P. M. Vanhove, Simona Georgieva

**Affiliations:** 1 Department of Botany and Zoology, Faculty of Science, Masaryk University, Kotlářská 2, 611 37 Brno, Czech Republic; 2 Centre for Environmental Sciences, Research Group Zoology: Biodiversity & Toxicology, Hasselt University, Agoralaan Gebouw D, 3590 Diepenbeek, Belgium; 3 Laboratory of Biodiversity and Evolutionary Genomics, Department of Biology, KU Leuven, Ch. Deberiotstraat 32, 3000 Leuven, Belgium; 4 Parasitic Worms Division, Department of Life Sciences, The Natural History Museum, Cromwell Road, London, SW7 5BD UK; 5 Institute of Biology, University of Graz, Universitätsplatz 2, 8010 Graz, Austria; 6 Zoology Unit, Finnish Museum of Natural History, University of Helsinki, P.O.Box 17, Helsinki, 00014 Finland; 7 Cavanilles Institute of Biodiversity and Evolutionary Biology, Science Park, University of Valencia, P.O. Box 46071, Valencia, Spain

**Keywords:** *Neocladocystis bemba* n. sp., *Neocladocystis biliaris* n. sp., *Tanganyikatrema* n. g., *Grandifundilamena* n. g., Species complex

## Abstract

**Background:**

Lake Tanganyika is considered a biodiversity hotspot with exceptional species richness and level of endemism. Given the global importance of the lake in the field of evolutionary biology, the understudied status of its parasite fauna is surprising with a single digenean species reported to date. Although the most famous group within the lake’s fish fauna are cichlids, the pelagic zone is occupied mainly by endemic species of clupeids (Actinopterygii: Clupeidae) and lates perches (Actinopterygii: Latidae, *Lates* Cuvier), which are an important commercial source for local fisheries. In this study, we focused on the lake’s four lates perches and targeted their thus far unexplored endoparasitic digenean fauna.

**Methods:**

A total of 85 lates perches from four localities in Lake Tanganyika were examined. Cryptogonimid digeneans were studied by means of morphological and molecular characterisation. Partial sequences of the nuclear *28S* rRNA gene and the mitochondrial cytochrome *c* oxidase subunit 1 (*cox*1) gene were sequenced for a representative subset of the specimens recovered. Phylogenetic analyses were conducted at the family level under Bayesian inference.

**Results:**

Our integrative approach revealed the presence of six species within the family Cryptogonimidae Ward, 1917. Three out of the four species of *Lates* were found to be infected with at least one cryptogonimid species. Two out of the three reported genera are new to science. Low interspecific but high intraspecific phenotypic and genetic diversity was found among *Neocladocystis* spp. Phylogenetic inference based on partial *28S* rDNA sequences revealed a sister group relationship for two of the newly erected genera and their close relatedness to the widely distributed genus *Acanthostomum* Looss, 1899.

**Conclusions:**

The present study provides the first comprehensive characterisation of the digenean diversity in a fish family from Lake Tanganyika which will serve as a baseline for future explorations of the lake’s digenean fauna. Our study highlights the importance of employing an integrative approach for revealing the diversity in this unique host-parasite system. 
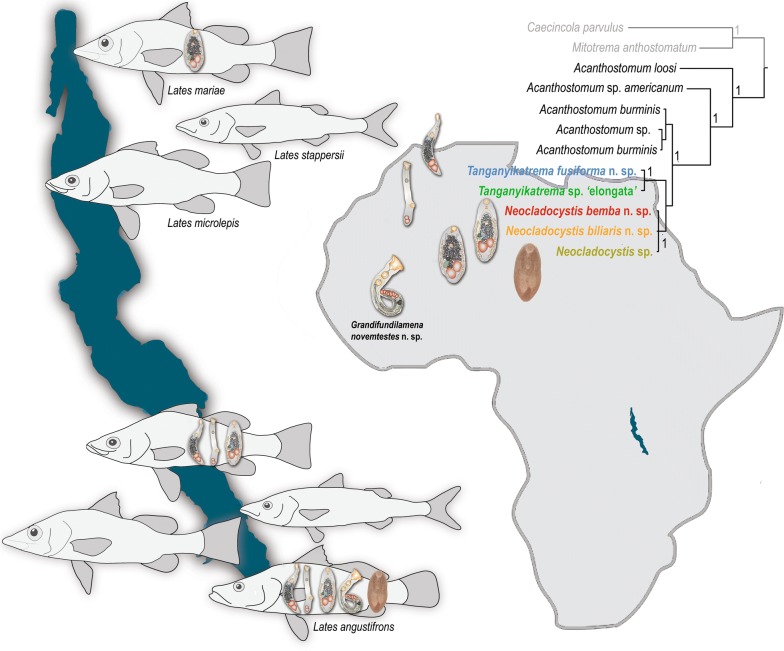

## Background

Lake Tanganyika, the oldest African rift lake (9–12 myr [1]), has attracted scientific exploration since the mid-nineteenth century and is recognised as an evolutionary reservoir and hotspot of diversification [2–4]. It is known for its great diversity of both vertebrate and invertebrate taxa. Compared to the other East African Great Lakes, Lake Tanganyika is characterised by the highest number of endemic species flocks with the greatest number of endemic non-cichlid fish species [5]. Together with the tremendous diversity of fishes, also a stunning diversity of parasites is known to have evolved in at least one cichlid fish lineage in the lake [6]. However, the current knowledge on the parasite diversity in Lake Tanganyika is rather limited. Only a negligible portion of the fish host species have been subjected to studies [7, 8] and so far, parasitological surveys have been mainly focused on cichlid fishes and their monogenean parasites [9–14]. Currently, a total of 59 helminth parasite species are described in the lake [7, 11–16]. One of the Lake Tanganyika’s smaller fish radiations are the four lates perches of the genus *Lates* Cuvier (Actinopterygii: Latidae), i.e. *Lates mariae* Steindachner, *Lates microlepis* Boulenger, *Lates angustifrons* Boulenger and *Lates stappersii* (Boulenger), important members of the pelagic and benthopelagic lake ecosystems [17, 18] and a commercial source for local fisheries [19]. Lates perches have lakewide distributions, a pattern seen also in other pelagic fish species in Lake Tanganyika such as clupeids and some cichlids [20–23]. All four *Lates* spp. are top predators in the lake’s open water; however, differentiation in habitat preferences can be detected [17].

Almost nothing is known on the digenean fauna infecting the endemic *Lates* spp. in Lake Tanganyika which is in contrast to the fairly large amount of data on *L. niloticus* (L.) and their Asian congener, *L. calcarifer* (Bloch) (see overview on the known helminth fauna of the latid family members in Table 1) [24–42]. The present knowledge on the parasite fauna of *Lates* spp. in the lake includes records for a single monogenean species infecting three out of the four species [16], and an unidentified larval nematode belonging to *Dujardinascaris* Baylis, 1947 from *L. microlepis* [15]. Currently, a single trematode species is known from the lake, i.e. *Neocladocystis tanganyikae* (Prudhoe, 1951) (Digenea: Cryptogonimidae) originally described by Prudhoe [43] as *Cladocystis tanganyikae* Prudhoe, 1951 found among a collection of several cichlid species and *Lamprichthys tanganicanus* (Boulenger). However, given the uncertainty of the host species, this record has to be revalidated [7].

**Table 1:** List of digenean species described from members of the family Latidae

Host	Digenean species	Family	Reference
*Lates niloticus* (L.)	*Euclinostomum* sp.	Clinostomidae	[24]
	*Acanthostomum knobus* Issa, 1962	Cryptogonimidae	[25, 26]
	*Acanthostomum niloticum* Issa, 1962		[25, 26]
	*Acanthostomum spiniceps* (Looss, 1896) Looss, 1899		[27]
	*Tylodelphys* sp. (metacercaria)	Diplostomidae	[28]
	*Echinostoma* sp.	Echinostomatidae	[29]
*Lates calcarifer* (Bloch)	*Stephanostomum cloacum* (Srivastava, 1938) Manter & Van Cleave, 1951	Acanthocolpidae	[30]
	*Allocreadium fasciatusi* Kakaji, 1969^a^	Allocreadiidae	[31]
	*Cardicola* sp.	Aporocotylidae	[32]
	*Cruoricola lates* Herbert, Shaharom-Harrison & Overstreet, 1994		[32, 33]
	*Parasanguinicola vastispina* Herbert & Shaharom, 1995		[32, 33]
	*Prosorhynchus luzonicus* Velasquez, 1959	Bucephalidae	[34]
	*Prosorhynchus* sp.		[35]
	*Callodistomum minutus* Zaidi & Khan, 1977	Callodistomidae	[36]
	*Pseudometadena celebesensis* Yamaguti, 1952	Cryptogonimidae	[34]
	*Pseudometadena* sp.		[34]
	*Proctoeces maculatus* (Looss, 1901) Odhner, 1911^b^	Fellodistomidae	[36]
	*Pseudohypertrema karachiense* Bilqees, 1976		[37]
	*Erilepturus hamati* (Yamaguti, 1934) Manter, 1947^c^	Hemiuridae	[38]
	*Lecithochirium* sp.		[34]
	*Opecoelus piriformis* Yamaguti, 1952	Opecoelidae	[39]
	*Psilostomum* sp.	Psilostomidae	[34]
	Sanguinicolidae gen. sp.	Sanguinicolidae	[35]
	*Prototransversotrema steeri* Angel, 1969	Transversotrematidae	[40]
	*Transversotrema patialense* (Soparkar, 1924) Crusz & Sathananthan, 1960		[34, 39]
*Psammoperca waigiensis* (Cuvier)	*Ningalooia psammopercae* Bray & Cribb, 2007	Acanthocolpidae	[42]

^a^ Reported as *Psilostomum chilkai* Chatterji, 1956

^b^ Reported as *Complexobursa magna* Bilqees, 1979

^c^ Reported as *Lecithochirium neopacificum* Velasquez, 1962

Members of the family Cryptogonimidae Ward, 1917 are parasitic in the intestine and/or pyloric caeca of marine and freshwater teleosts, reptiles and amphibians [44]. Of the over 200 species of 64 genera reported worldwide [44] only seven species of four genera, *Acanthostomum* Looss 1899, *Brientrema* Dollfus, 1950, *Neocladocystis* Manter & Ritchard, 1696 and *Siphodera* Linton, 1910, are known from African freshwater fishes [25, 26, 43, 45–49]. This emphasises the lack of research and constrains further parasitological studies on the ecology, evolution and conservation of economically important fish species.

The present study aims to increase the knowledge on the parasite fauna of the economically important lates perches, i.e. *L*. *angustifrons*, *L*. *mariae*, *L*. *microlepis* and *L. stappersii* endemic to Lake Tanganyika, and particularly on the digenean trematodes as an integral component of the local food chain and the ecosystem [50]. Here, we provide the first molecular data for trematode parasites from the lake accompanied by morphological characterisation and descriptions. Additionally, phylogenetic inference based on DNA sequence data is used to evaluate the phylogenetic relationships of the newly described species at family level.

## Methods

### Collection and fixation of specimens

**Fig. 1:**
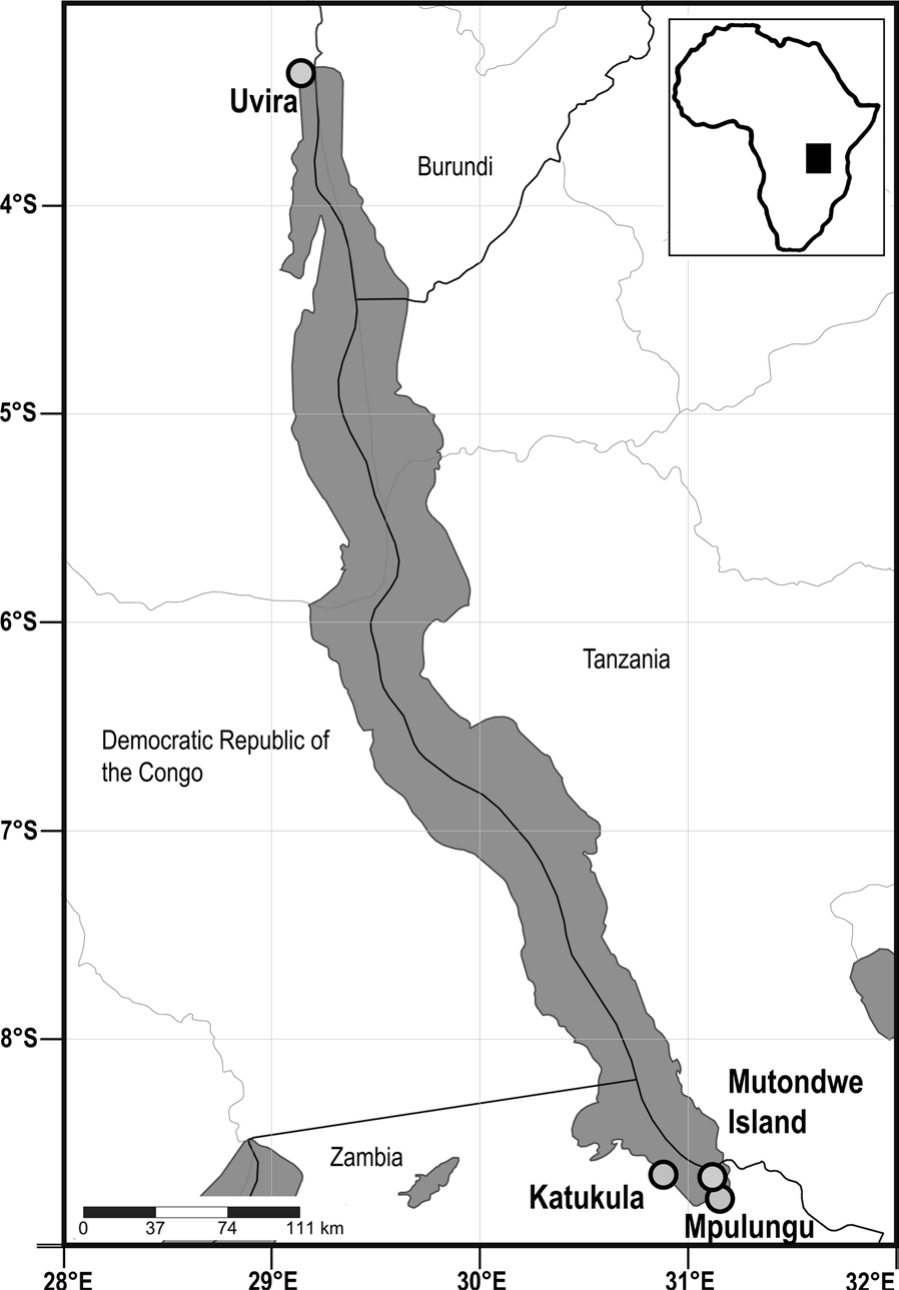
Map of Lake Tanganyika with the sampling locations of *Lates* spp. The map was created using SimpleMappr software v7.0.0. (http://www.simplemappr.net). Accessed 5 Mar 2019

Specimens of four species of *Lates*, *L. angustifrons*, *L. mariae*, *L. microlepis* and *L. stappersii*, were either purchased from local fishermen or collected by hand nets during field trips in 2016 and 2018, respectively (see Table 2) [51]. A total of 85 specimens was sampled at four sampling locations: (i) at the northern part of the lake off Uvira, Democratic Republic of the Congo; and (ii) at the southern part of the lake at Katukula, Mpulungu (fish market) and Mutondwe Island (all three in Zambia; see Fig. 1 for further details). Fish were examined fresh following the standard protocol of Ergens & Lom [52]. The recovered digenean trematodes were rinsed and cleaned in a Petri dish with saline solution; most of the saline solution was gently removed by pipetting and the specimens were killed by nearly boiling water. Subsequently, the trematode specimens were preserved either in 4% formalin and 70% ethanol, or in 96% ethanol for morphological and molecular studies, respectively.

**Table 2:** Distribution and infection parameters of cryptogonimid species recovered in this study

Host species	Locality	Coordinates	Locality sub-basin^a^	Date of collection	No. of fish specimens examined	No. of fish specimens infected^b^
*L. angustifrons*	Mpulungu	8°46ʹS, 31°07ʹE	Southern	12.iv.2018	7	2/0/1/2/1/1
*L. mariae*	Uvira	3°22ʹS, 29°09ʹE	Northern	12.viii.2016	2	0/1/0/0/0/0
	Mpulungu	8°46ʹS, 31°07ʹE	Southern	16.iv.2018	11	0/0/0/0/0/0
*L. microlepis*	Mutondwe Island	8°42ʹS, 31°07ʹE	Southern	16.iv.2018	8	3/0/0/2/0/0
	Katukula	8°43ʹS, 30°57ʹE	Southern	14.iv.2018	5	3/0/0/3/0/0
	Mpulungu	8°46ʹS, 31°07ʹE	Southern	13.iv.2018	14	8/0/0/3/2/0
	Uvira	3°22ʹS, 29°09ʹE	Northern	12.viii.2016	7	0/0/0/0/0/0
*L. stappersii*	Mpulungu	8°46ʹS, 31°07ʹE	Southern	6.iv.2018	3	0/0/0/0/0/0
	Uvira	3°22ʹS, 29°09ʹE	Northern	12.viii.2016	28	0/0/0/0/0/0

^a^ After Danley et al. [51]

^b^ Infection parameters are provided in the following order: *Neocladocystis bemba* n. sp./*Neocladocystis biliaris* n. sp./*Neocladocystis* sp./*Tanganyikatrema fusiforma* n. sp./*Tanganyikatrema* sp. ‛elongataʼ/*Grandifundilamena novemtestes* n. sp

### Morphological examination

Specimens preserved in 4% formalin or 70% ethanol were stained with iron acetocarmine, dehydrated through a graded ethanol series, cleared in dimethyl phthalate and examined as permanent mounts in Canada balsam. All specimens for which sequence data were generated were preserved in 96% ethanol and later photographed from wet mounts in distilled water using the Leica Application Suite v.4.3.0. analysis software on a Leica DMR light microscope (Wetzlar, Germany) at magnifications of 100–1000×. Subsequently, a piece of the posterior part of the specimen (the post-testicular region) was excised and used for DNA isolation. The remaining anterior part of the specimen was kept as molecular voucher (i.e. hologenophores *sensu* Pleijel et al. [53]).

Specimens prepared as whole mounts were both photographed and drawn using a drawing tube at a high magnification. Measurements were taken from photomicrographs using the Leica Application Suite v.4.3.0. analysis software. In total, 33 different characters were measured and the following abbreviations were used: BL, body length; BW, body width; OSL, oral sucker length; OSW, oral sucker width; VSL, ventral sucker length; VSW, ventral sucker width; FBL, forebody length; HBL, hindbody length; PPH, prepharynx length; PHL, pharynx length; PHW, pharynx width; OL, oesophagus length; IB-F, distance from anterior extremity to intestinal bifurcation; IB-VS, distance from intestinal bifurcation to ventral sucker; POSTCL, length of post-caecal field; ATL, anterior testis length; ATW, anterior testis width; PTL, posterior testis length; PTW, posterior testis width; POSTL, length of post-testicular field; OVL, ovary length; OVW, ovary width; ABE-OV, distance from anterior body extremity to ovary; VS-OV, distance from ventral sucker to ovary; OV-AT, distance from ovary to anterior testis; EL, egg length; EW, egg width; POSTUL, length of post-uterine field; PREVIL, length of pre-vitelline field; VITL, length of vitelline field; POSTVITL, length of post-vitelline field; SRL, length of seminal receptacle; SRW, width of seminal receptacle. Further, the following ratios were measured VSL/OSL, sucker length ratio; VSW/OSW, sucker width ratio; OSL/BL (%), oral sucker as a proportion of body length; VSL/BL (%), ventral sucker length as a proportion of body length; FBL/BL (%), forebody length as a proportion of body length; HBL/BL (%), hindbody length as a proportion to body length; PHL/BL (%), pharynx length as a proportion of body length; IB-F/BL (%), length of pre-intestinal field as a proportion of body length; POSTCL/BL (%), length of post-caecal field as a proportion of body length; ATL/BL (%), anterior testis length as a proportion of body length; PTL/BL (%), posterior testis length as a proportion of body length; POSTL/BL (%), post-testicular field length as a proportion of body length; ABE-OVL/BL (%), anterior body extremity to ovary distance as a proportion of body length; VS-OV/BL (%), distance from ventral sucker to ovary as a proportion of body length; OV-AT/BL (%), distance from ovary to anterior testis as a proportion of body length; OV/BL (%), ovary length as a proportion of body length; POSTUL/BL (%), length of post-uterine field as a proportion of body length; PREVITL/BL (%), length of pre-vitelline field as a proportion of body length; VITL/BL (%), length of vitelline field as a proportion of body length; POSTVITL/BL (%), length of post-vitelline field as a proportion of body length; SRL/BL (%), seminal receptaculum length as a proportion of body length; BW/BL (%), body width as a proportion of body length. The terminology of the measured characters follows Miller & Cribb [44]. The type- and voucher material is deposited at the Helminthological Collection of the Natural History Museum, London, UK (NHMUK) and in the collection of the Research Group Zoology: Biodiversity and Toxicology at Hasselt University in Diepenbeek, Belgium (HU).

To comply with the regulations set out in article 8.5 of the amended 2012 version of the *International Code of Zoological Nomenclature* (ICZN [54]), details of the species have been submitted to ZooBank. The Life Science Identifier (LSID of the article is urn:lsid:zoobank.org:pub:9C751425-E16A-4E21-82A6-EB0016FA3899). For each new taxon, the LSID is reported in the taxonomic summary.

### Molecular data generation

The posterior portion in cases of larger specimens or complete specimens in cases of very small specimens were used for genomic DNA isolation. Total genomic DNA (gDNA) isolation was performed with a 5% Chelex® suspension and 0.2 mg/ml of proteinase *K* (see Dallarés et al. 2013 for details [55]). Partial DNA sequences were generated for both *28S* ribosomal RNA (rRNA) gene (domains D1-D3) and the mitochondrial cytochrome *c* oxidase subunit 1 (*cox*1) gene. PCR amplification was carried out using the primer combinations digl2 (forward: 5ʹ-AAG CAT ATC ACT AAG CGG-3ʹ) or LSU5 (forward: 5ʹ-TAG GTC GAC CCG CTG AAY TTA AGC A-3)ʹ and 1500R (reverse: 5ʹ-GCT ATC CTG AGG GAA ACT TCG-3ʹ) (Tkach et al. [56]) in the case of *28S* rDNA and JB3 (forward: 5ʹ-TTT TTT GGG CAT CCT GAG GTT TAT-3ʹ; Bowles et al. [57]) and CO1-R trema (reverse: 5ʹ-CAA CAA AAT CAT GAT GCA AAA GG-3ʹ; Miura et al. [58]) in the case of *cox*1. Amplification reactions were performed in a total volume of 20 µl using 2× MyFi™ DNA Polymerase Mix (Bioline Inc., Taunton, USA), and *c*.50 ng of gDNA. PCR reactions were performed under the following thermocycling conditions: (i) *28S*: initial denaturation at 95 °C for 5 min followed by 40 cycles of 95 °C for 30 s, annealing at 55 °C for 30 s, extension at 72 °C for 2 min, and a final extension step at 72 °C for 7 min; (ii) *cox*1: initial denaturation at 95 °C for 3 min followed by 35 cycles of 95 °C for 50 s, annealing at 50 °C for 50 s, extension at 72 °C for 2 min and a final extension step at 72 °C for 7 min. PCR products were purified using QIAquick PCR purification kit (Qiagen Ltd., Hilden, Germany). Both strands were cycle-sequenced using the ABI BigDye™ 3.1 Chemistry (ABI Perkin-Elmer, London, UK) on a 3730xl DNA Analyser (ABI Perkin-Elmer, London, UK) at GATC Biotech (Konstanz, Germany). The PCR primers and an additional internal primer 300F (forward: 5ʹ-CAA GTA CCG TGA GGG AAA GTT G-3ʹ; Littlewood et al. [59]) in the case of *28S* rDNA were used for the sequencing reactions. Contiguous sequences were assembled using Geneious v.8 (http://www.geneious.com/; Kearse et al. [60]) and submitted to the GenBank database under the accession numbers MN705808-MN705812 (*28S* rDNA) and MN702809-MN702817 (*cox*1) (see Table 3 for provenance data and GenBank accession numbers).

**Table 3:** GenBank accession numbers for sequences (*28S* rRNA gene and *cox*1 gene) for the digenean species generated in the present study

Parasite species	Host species	Locality	GenBank ID	
			*28S* rRNA	*cox*1
*Neocladocystis bemba* n. sp.	*L. microlepis*	Katukula (8°43ʹS, 30°57ʹE)	MN705808	MN702809, MN702812, MN702814, MN702815
		Mpulungu (8°46ʹS, 31°07ʹE)		MN702813
		Mutondwe Island (8°42ʹS, 31°07ʹE)		MN702810, MN702811
*Neocladocytis biliaris* n. sp.	*L. mariae*	Uvira (4°20ʹS, 29°09ʹE)	MN705809	
*Neocladocytis* sp.	*L. microlepis*	Mutondwe Island (8°42ʹS, 31°07ʹE)	MN705810	MN702816
*Tanganyikatrema fusiforma* n. sp.	*L. microlepis*	Mpulungu (8°46ʹS, 31°07ʹE)	MN705811	MN702817
*Tanganyikatrema* sp. ‛elongataʼ	*L. angustifrons*	Mpulungu (8°46ʹS, 31°07ʹE)	MN705812	

### Sequence alignments and phylogenetic analyses

Two main alignments for the partial *28S* rDNA data, including selected sequences downloaded from GenBank (see Additional file 1: Table S1), were built to infer the phylogenetic position of the African cryptogonimids: (i) a set of 52 taxa of the Cryptogonimidae Ward, 1917 (843 bp); and (ii) a restricted dataset of 10 species of *Acanthostomum* Looss, 1899 (489 bp). Sequences were aligned in MAFFT v.7 [61] on the EMBL-EBL bioinformatics web platform (http://www.ebi.ac.uk/Tools/msa/mafft/) under the default settings with a gap opening penalty of 1.53 and a gap extension penalty of 0.123 over 1000 cycles of iterative refinement incorporating local pairwise alignment information [61]. Highly variable parts of the alignments were determined and excluded by Gblocks [62] as implemented in SeaView v.4 [63] under less stringent parameters and refined by eye. Uncorrected pairwise distances were calculated in MEGA v.7 [64]. jModelTest v.2 [65] was used to select the best-fitting models of sequence evolution under the Bayesian information criterion.

Phylogenetic relationships were inferred under Bayesian inference (BI) in MrBayes v3.2.0 [66]. Two independent runs were performed for 10,000,000 generations and sampled every 1000th generation. The ‛burn-inʼ was set for the first 25% of the sampled trees. Bayesian analyses were executed online on the CIPRES Science Gateway v. 3.3 [67]. Parameter convergence and run stationarity were assessed in Tracer v1.6 [68]. The outgroup choices were informed by broader phylogenies of the Digenea [69]. The resulting trees were visualised in FigTree v.1.4.3 (http://tree.bio.ed.ac.uk/software/figtree/). All species included in the phylogenetic analyses together with their GenBank accession numbers are listed in Additional file 1: Table S1.

The *cox*1 sequence alignment comprised only newly generated sequences for three of the cryptogonimids recovered from Lake Tanganyika lates perches. The examined matrix consisted of 795 bp of nine terminals.

## Results

### Digenean diversity in Lake Tanganyika’s lates perches

Examination of 85 individuals of lates perches from four localities in Lake Tanganyika (all four endemic species were included in our dataset, Table 2) revealed a total of 32 infections with cryptogonimid trematodes. Three out of the four *Lates* species examined (i.e. *L. angustifrons*, *L. mariae* and *L. microlepis)*, were infected with at least one species of cryptogonimid digenean. No digeneans were recovered from *L. stappersii* in neither of the two localities where the species had been sampled. Distribution and infection parameters are listed in Table 2. Adult cryptogonimids were detected in the fish intestine, pyloric caeca and gall-bladder while immature specimens were recovered only in the intestine. Sequence data were successfully generated for representatives of five out of the six species recovered. The newly recovered cryptogonimids exhibited specific morphological and molecular features when compared with other members of the family. The taxonomy proposed here is based on a combined morphological and molecular approach which resulted in the description of six new species and the erection of two new genera.

**Taxonomy**

**Superfamily Opisthorchioidea Looss, 1899**

**Family Cryptogonimidae Ward, 1917**

**Genus*Neocladocystis* Manter & Pritchard, 1969**

***Amended diagnosis***

Based on Manter & Pritchard (1969 [48]). Body oval, widest at level of ventral sucker, length:width ratio 1.5–3.0. Oral sucker subterminal, spherical. Circumoral spines lacking. Tegumental spines present or absent. Ventral sucker unspecialised, small, pre-equatorial, not obviously embedded in ventrogenital sac. Sucker-width ratio *c*.1.7. Forebody 25–35% of body length. Prepharynx short, narrow. Oesophagus short or indistinguishable. Intestinal bifurcation immediately anterior to ventral sucker. Caeca blind, narrow, end at level of testes. Testes two, symmetrical to slightly oblique, at posterior extremity of body. Seminal vesicle elongate-oval, naked. Gonotyl absent. Ovary lobed, just anterior to testes. Uterus in hindbody, between testes and ventral sucker, may extend to intestinal bifurcation in mid-forebody. Vitellarium follicular; vitelline follicles in two lateral fields, mainly in hindbody, from level of testes to ventral sucker, may reach level of intestinal bifurcation. Arms of excretory vesicle may almost reach level of pharynx, sometimes do not exceed intestinal bifurcation. In intestine and gall-bladder of freshwater fishes (Cichlidae, Characidae, Bagridae, Latidae); Africa and South America. *Type-species*: *Neocladocystis tanganyikae* (Prudhoe, 1951) Manter & Pritchard, 1969.

**Differential diagnosis**

Species of *Neocladocystis* morphologically and genetically resemble members of *Acanthostomum* Looss, 1899, which are cosmopolitan parasites in fishes and reptiles. Morphological similarities include a round oral sucker opening subterminally and a short oesophagus. They differ in possessing blind caeca and the absence of circumoral spines. *Brientrema* Dollfus, 1950 with members infecting freshwater fishes (Malapteruridae, Citharinidae) in Africa resembles *Neocladocystis* by the possession of a nearly spherical oral sucker, a very short prepharynx and oesophagus, blind caeca and two slightly oblique testes. However, the two genera differ in the presence *versus* absence of circumoral spines and in vitelline fields reaching about the level of the ventral sucker in *Neocladocystis*.

***Neocladocystis bemba*Georgieva, Kmentová & Bray n. sp.**

***Type-host*:***Lates microlepis* Boulenger (Actinopterygii: Latidae).

***Other host*:***Lates angustifrons* Boulenger (Actinopterygii: Latidae).

***Type-locality*:** Lake Tanganyika at Mpulungu (8°46ʹS, 31°07ʹE), Zambia.

***Other localities*:** Lake Tanganyika at Mutondwe Island (8°42ʹS, 31°07ʹE) and Katukula (8°36ʹS, 31°11ʹE), Zambia.

***Type-specimens*:** The holotype (NHMUK.2019.11.18.1) and 8 paratypes (NHMUK.2019.11.18.2-9) were deposited in the Helminthological Collection of the Natural History Museum, London, UK, and 10 paratypes (HU 760-69) were deposited in the Collection of Hasselt University, Diepenbeek, Belgium.

***Site in host*:** Immature specimens in intestine and egg-bearing specimens in pyloric caeca.

***Representative DNA sequences*:** GenBank: MN705808 (partial *28S* rRNA gene, domains D1-D3); MN702809-MN702815 (*cox*1).

***ZooBank registration*:** The LSID for the new name *Neocladocystis bemba* is urn:lsid:zoobank.org:act:30402449-A3C5-48FA-A700-25063221630B.

***Etymology*:** The species is named after the language spoken by a large part of the Zambian population.

**Description**

**Fig. 2:**
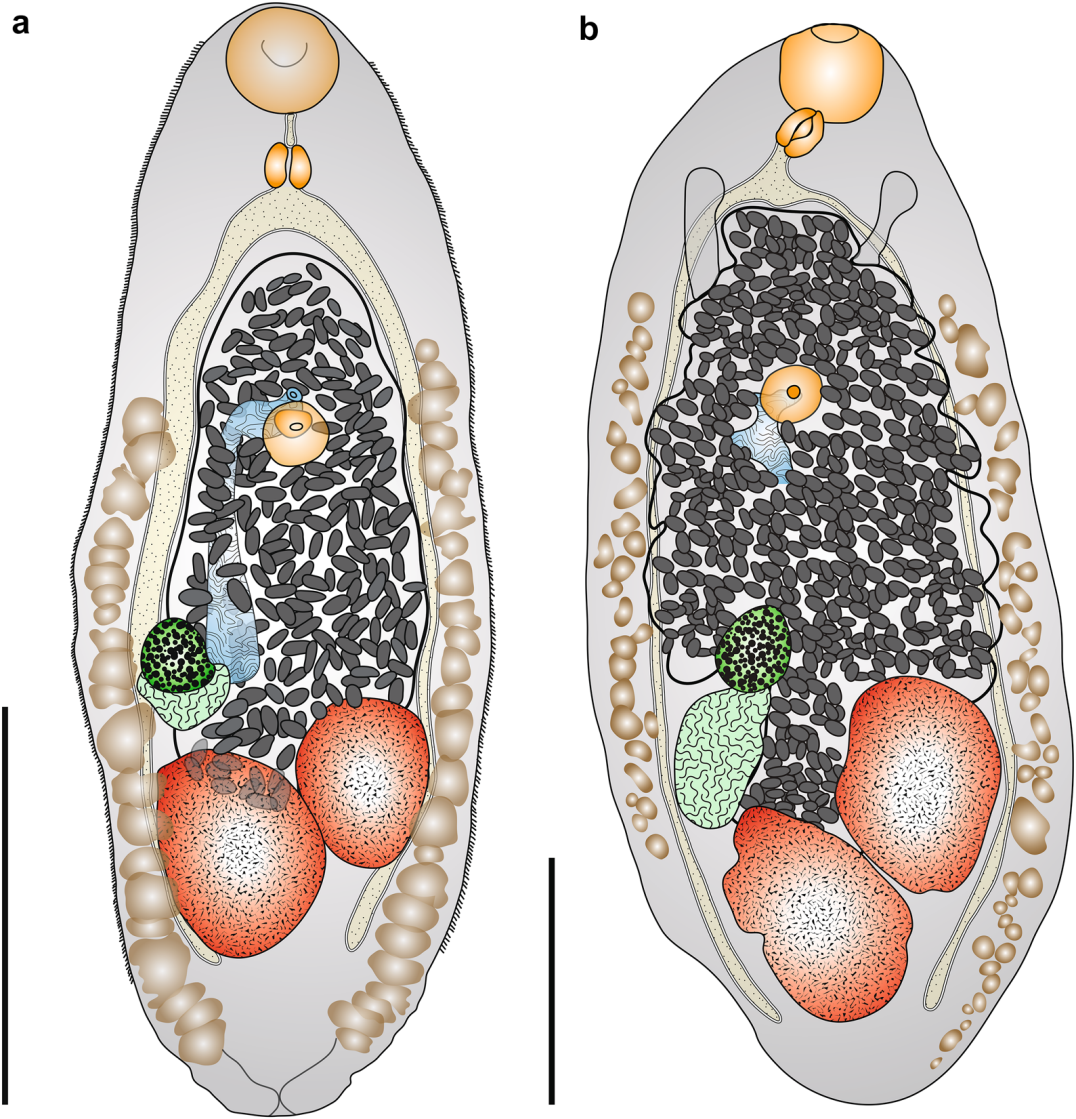
Line drawings of paragenophores of *Neocladocystis* spp. **a***Neocladocystis bemba* n. sp. from the pyloric caeca of *Lates microlepis* off Mutondwe Island, Lake Tanganyika. **b***Neocladocystis biliaris* n. sp. from the liver of *L. mariae*, Uvira fish market, Lake Tanganyika. *Scale-bars*: 500 µm

[Based on 21 specimens including 6 immature individuals; Fig. 2a, Table 4, Additional file 2: Figure S1.] Body irregularly oval, flattened. Tegument spined; spines reach close to posterior extremity, posterior to level of caeca, largest at mid-body level. Oral sucker spherical, subterminal. Ventral sucker pre-equatorial, rounded, may be completely obscured by eggs, distinctly smaller than oral sucker. Prepharynx short or undistinguishable. Pharynx oval, muscular, longer than wide. Oesophagus often not detectable, occasionally short. Intestinal bifurcation in about mid-forebody. Caeca relatively wide, reach into post-testicular region.

**Table 4:** Comparative morphometric data for the newly described species of *Neocladocystis*, *Tanganyikatrema* n. g. and *Grandifundilamena novemtestes* n. g.

	*Neocladocystis bemba* n. sp.	*Neocladocystis bemba* n. sp. (immature)	*Neocladocystis biliaris* n. sp.	*Tanganyikatrema fusiforma* n. sp.	*Tanganyikatrema fusiforma* n. sp. (immature)	*Tanganyikatrema* sp. *‛*elongataʼ	*Grandifundilamena novemtestes* n. sp.
BL	1067–1823 (1329)^a^	255–1028 (597)^j^	1940–3074 (2286)^j^	632–1574 (990)^j^	335–525 (456)^m^	437–679 (558)^n^	3458–4585 (3948)^m^
BW	475–745 (609)^a^	114–401 (273)^j^	944–1201 (1000)^j^	146–387 (249)^j^	103–171 (133)^m^	95–96 (95.5)^n^	303–414 (362)^m^
OSL	114–176 (141)^b^	81–160 (99)^j^	198–272 (225)^g^	119–206 (157)^j^	100–115 (108)^m^	107–110 (108.5)^n^	355–482 (415)^m^
OSW	128–189 (154)^b^	68–168 (108)^j^	206–269 (231)^g^	70–152 (115)^i^	65–77 (73)^m^	69–92 (80)^m^	425–612 (510)^m^
VSL	71–114 (92)^c^	47–84 (57)^j^	93–121 (109)^m^	62–134 (105)^i^	51–84 (64)^m^	54–58 (56)^n^	165–206 (187)^m^
VSW	75–124 (98)^d^	51–81 (57)^j^	106–136 (121)^m^	54–127 (107)^i^	54–91 (68)^m^	53–54 (53.5)^n^	168–216 (189)^m^
FBL	349–514 (422)^c^	83–369 (207)^g^	679–698 (689)^n^	220–494 (306)^i^	128–237 (189)^m^	262–419 (304.5)^n^	787–1063 (927)^m^
HBL	719–1210 (874)^c^	125–575 (333)^g^	1140–1408 (1274)^n^	320–765 (510)^d^	156–253 (205)^m^	202–209 (205.5)^n^	2506–3316 (2883)^m^
PPH	10–38 (18)^j^	not detectable	15–20 (18)^n^	20–122 (53)^l^	not detectable	80–120 (100)^n^	80–264 (170)^m^
PHL	61–96 (70)^e^	38–73 (54)^g^	93–107 (98)^l^	64–122 (87)^c^	35^o^	39–50 (45)^n^	223–278 (247)^m^
PHW	47–94 (60)^e^	34–56 (46)^l^	79–89 (86)^l^	35–76 (52)^c^	39^o^	25–29 (27)^n^	160–224 (189)^m^
OL	5–38 (18) ^j^	not detectable	15–20 (18)^l^	43–100 (74)^m^	not detectable	60^o^	absent
IB-F	159–271 (223)^a^	70–132 (103)^l^	313–364 (341)^l^	146–412 (270)^c^	148–222 (185)^n^	270^o^	632–820 (734)^m^
IB-VS	201–249 (223)^g^	50–95 (68)^g^	313–346 (330)^n^	0–82 (45)^g^	0–13 (6.5)^n^	0^o^	121–265 (189)^m^
POSTCL	89–250 (158)^a^	26–72 (55)^g^	116–303 (248)^g^	18–59 (36)^c^	12–60 (36)^n^	–	53–101 (77)^m^
ATL	181–326 (237)^f^	100–206 (139)^g^	287–430 (379)^j^	82–183 (129)^j^	38–66 (52)^n^	53^o^	81–160 (121)^m^
ATW	135–255 (183)^f^	78–113 (98)^g^	278–359 (304)^j^	75–214 (143)^d^	56–66 (61) ^n^	47^o^	99–176 (141)^m^
PTL	167–394 (254)^h^	95–197 (138)^g^	348–450 (416)^j^	99–171 (136)^c^	37–65 (51)^n^	5^o^	120–160 (146)^m^
PTW	135–306 (226)^e^	67–128 (90)^g^	248–416 (309)^j^	88–187 (127)^c^	56–71 (63.5)^n^	58^o^	95–157 (130)^m^
POSTL	132–401 (205)^h^	67–162 (110)^g^	80–323 (201)^g^	44–126 (85)^c^	16–22 (19)^n^	29^o^	55–209 (128)^m^
OVL	140–296 (176)^c^	70–113 (92)^g^	213–284 (242)^m^	47–120 (83)^i^	28–66 (46)^m^	–	166–196 (181)^m^
OVW	144–247 (185)^i^	65–109 (81)^g^	186–319 (241)^l^	53–102 (73)^i^	41–47 (43)^m^	–	102–177 (140)^m^
ABE-OV	510–720 (608)^c^	243–296 (261)^l^	1073–1868 (1344)^m^	487–1124 (695)^c^	209^o^	–	1978–3355 (2676)^m^
VS-OV	73–200 (145)^j^	8–78 (28)^g^	295^o^	120–496 (257)^i^	30–41 (35.5)^n^	–	1026–2077 (1560)^m^
OV-AT	65–220 (123)^j^	30–80 (58)^g^	113–173 (141)^l^	0^i^	0^n^	–	100–355 (220)^m^
EL	34–40 (37)^e^	not detectable	33–38 (35,3)^d^	27–39 (31)^c^	not detectable	29^o^	26–28 (27)^m^
EW	12–19 (16)^e^	not detectable	13–18 (15,9)^d^	13–20 (16)^c^	not detectable	15^o^	10–11 (10.5)^m^
POSTUL	176–478 (353)^f^	120–300 (203)^g^	457–683 (587)^j^	171–397 (281)^c^	79–140 (115.3)^m^	–	636–828 (732)^m^
PREVIL	228–455 (318)^f^	not detectable	139–578 (447)^j^	320–583 (396)^d^	280^o^	–	1879–2208 (2043)^m^
VITL	714–1206 (944)^k^	not detectable	1356–1648 (1518)^l^	226–483 (359)^d^	120^o^	–	2100–2200 (2160)^m^
POSTVITL	59–150 (96)^k^	not detectable	80–213 (134)^d^	198–603 (355)^c^	125^o^	–	166–310 (229)^m^
SRL	79–220 (139)^j^	not detectable	205–323 (264)^j^	52–156 (86)^l^	40–41 (40.5)^n^	–	129–214 (172)^m^
SRW	82–184 (136)^d^	not detectable	119–251 (173)^j^	42–98 (59)^l^	18–33 (25.5)^n^	–	156–224 (190)^m^
VSL/OSL	1.2–2.1 (1.6)^c^	1.5–1.9 (1.7)^j^	1.7–1.8 (1.7)^n^	1.2–1.9 (1.5)^i^	1.4–2.2 (1.8)^m^	1.7–2.0 (1.9)^n^	2.15–2.3 (2.2)^m^
VSW/OSW	1.5–1.8 (1.7)^d^	1.3–2.1 (1.9)^j^	1.5–1.9 (1.7)^n^	0.8–1.4 (1.1)^i^	0.8–1.3 (1.1)^m^	1.5–1.7 (1.6)^n^	2.5–2.9 (2.7)^m^
OSL/BL (%)	8.1–12.7 (10.8)^a^	12.7–32.5 (18.5)^j^	8.8–11.0 (9.7)^g^	10.2–22.2 (17.2)^i^	19.7–32.8 (24.8)^m^	15.8–22.9 (19.3)^n^	10.2–10.5 (10.4)^m^
VSL/BL (%)	5.4–8.0 (6.6)^c^	6.9–18.4 (10.7)^j^	4.6–6.2 (5.3)^m^	6.0–19.1 (12.6)^i^	11.4–16.0 (14.2)^m^	7.9–13.3 (10.6)^n^	12.3–13.3 (12.8)^m^
FBL/BL (%)	26.0–35.4 (30.7)^c^	28.7–38.5 (34.6)^g^	31.2–35.0 (33.1)^n^	23.0- 40.0 (31.3)^i^	38.2–46.7 (41.0)^m^	59.9–61.7 (60.8)^n^	22.8–24.2 (23.2)^m^
HBL/BL (%)	57.6–67.2 (62.7)^c^	49.0–64.4 (54.4)^g^	58.8–62.9 (60.8)^n^	50.6–65.5 (57.7)^d^	40.8–48.2 (45.1)^m^	29.7–47.8 (38.8)^n^	71.7–72.3 (72.1)^m^
PHL/BL (%)	4.0–6.6 (5.3)^a^	5.2–11.8 (8.5)^g^	4.2–5.0 (4.6)^l^	5.7–16.3 (9.5)^c^	10.4^o^	5.7^o^	6.1–6.4 (6.2)^m^
IB-F/BL (%)	12.1–21.0 (16.22)^i^	22.1–25.1 (23.6)^m^	14.2–18.2 (15.9)^l^	11.3–42.8 (27.8)^j^	43.7–44.2 (43.9)^n^	–	17.9–18.3 (18.4)^m^
POSTCL/BL (%)	8.3–15.6 (11.9)^k^	5.0–14.2 (10.8)^g^	6–12.7 (10.5)^g^	2.2–7.9 (4.2)^c^	3.6–11.4 (7.5)^n^	–	1.5–2.2 (1.9)^m^
ATL/BL (%)	15.2–23.2 (18.5)^i^	18.2–22.9 (20.9)^g^	14.9–21.3 (18.8)^g^	9.6–14.1 (11.5)^j^	11.3–13.0 (12.2)^n^	7.8^o^	2.3–3.5 (3.0)^m^
PTL/BL (%)	15.0–28.0 (19.5)^k^	19.2–24.4 (20.7)^g^	17.5–22.3 (20.2)^g^	9.9–14.6 (12.5)^c^	14.0–16.7 (15.3)^n^	8.5^o^	3.0–4.6 (3.7)^m^
POSTL/BL (%)	12.6–22.0 (16.2)^a^	12.8–20.4 (16.7)^g^	4.0–14.4 (9.2)^g^	10.9–17.4 (13.5)^c^	11.0–12.8 (11.9)^n^	7.4^o^	1.6–4.6 (3.2)^m^
ABE-OV/BL (%)	39.0–54.9 (47.0)^c^	40.3–49.5 (45.9)^l^	48.5–60.8 (54.9)^m^	57.1–71.4 (66.9)^c^	62.4^o^	–	57.2–73.2 (68.2)^m^
VS-OV/BL (%)	6.3–14.5 (10.9)^j^	1.6–7.6 (3.5)^g^	15.2^o^	19.0–31.5 (25.1)^i^	8.1–9.0 (8.5)^n^	–	29.7–45.3 (40.0)^m^
OV-AT/BL (%)	5.3–19.2 (9.6)^j^	9.1–16.2 (11.6)^m^	5.0–7.9 (6.5)^l^	0^c^	0^m^	–	2.9–7.7 (5.2)^m^
OVL/BL (%)	10.7–25.9 (14.0)^d^	11.0–16.2 (14.1)^g^	9.2–11.9 (10.2)^m^	9.9–14.6 (12.5)^c^	14.0–16.7 (15.3)^n^	8.5^o^	4.3–4.8 (4.6)^m^
POSTUL/BL (%)	20.6–36.7 (28.2)^a^	24.3–31.0 (27.6)^n^	22.2–31.0 (26.3)^g^	23.5–34.6 (28.2)^d^	23.6–26.7 (25.1)^m^	–	18.1–18.4 (18.5)^m^
PREVITL/BL (%)	16.8–28.8 (21.9)^k^	not detectable	16.0–26.7 (22.3)^g^	31.2–46.4 (38.0)^d^	53.3^o^	–	47.6–48.2 (51.1)^m^
VITL/BL (%)	65.1–77.1 (70.3)^k^	not detectable	65.8–74.9 (70.4)^l^	29.9–55.3 (34.9)^j^	22.9^o^	–	48.4–53.2 (54.0)^m^
POSTVITL/BL (%)	5.5–10.0 (7.3)^i^	not detectable	3.2–11.0 (6.5)^j^	23.6–39.8 (35.4)^d^	23.8^o^	–	4.8–6.8 (5.9)^m^
SRL/BL (%)	7.4–15.5 (10.9)^g^	not detectable	10.2–14.7 (12.8)^g^	9.9–12.1 (11.0)^n^	not detectable	–	2.8–5.4 (4.3)^m^
BW/BL (%)	1.7–2.7 (2.2)^a^	1.9–2.6 (2.2)^j^	2.1–2.6 (2.3)^g^	3.2–5.7 (4.0)^i^	3.1–4.0 (3.4)^m^	4.6–7.1 (5.0)^n^	10.4–11.6 (11.1)^m^

*Notes:*^a^ (*n* = 11); ^b^ (*n* = 15); ^c^ (*n* = 8); ^d^ (*n* = 7); ^e^ (*n* = 14); ^f^ (*n* = 12); ^g^ (*n* = 5); ^h^ (*n* = 13); ^i^ (*n* = 9); ^j^ (*n* = 6); ^k^ (*n* = 10); ^l^ (*n* = 4); ^m^ (*n* = 3); ^n^ (*n* = 2); ^o^ (*n* = 1)

Testes 2, entire or slightly lobed, oblique, contiguous or slightly separated, in posterior half of hindbody. Seminal vesicle elongate-oval, dextral, naked, between ovary and ventral sucker. Gonotyl absent. Genital pore median, just anterior to ventral sucker.

Ovary small, subspherical or irregular, entire or slightly lobed, dextral, intercaecal, pre-testicular at distance from posterior testis. Mehlis’ gland and Laurer’s canal not observed. Seminal receptacle spherical or saccular, post-ovarian, immediately posterior to ovary or partially overlapping it dorsally. Uterine coils extend from level of testes to intestinal bifurcation, mostly intercaecal. Eggs numerous, noticeably variable, tanned, operculate. Vitellarium follicular, in 2 lateral fields, extend anteriorly from about level of ventral sucker to post-testicular field close to posterior extremity of body, overlapping caeca dorsally and ventrally. Seminal receptacle saccular, dorsal, post-ovarian.

Excretory vesicle Y-shaped, bifurcates just posterior to ventral sucker (seen in immature specimens). Excretory vesicle narrower posteriorly, widens and reaches at least to uterus, may reach anteriorly to about level of pharynx. Excretory pore terminal.

**Remarks**

Currently, only two species of *Neocladocystis* are known from Africa, i.e. *N. tanganyikae* (Prudhoe, 1951) Manter & Pritchard, 1969 and *N. congoensis* Manter & Pritchard, 1969. *Neocladocystis tanganyikae* was described from “residus de fixations des poissons” from Lake Tanganyika. These fishes were caught in a small bay south of Cape Tembwe on the Congolese side of Lake Tanganyika and apparently included *Lamprichthys tanganicanus* and several species of cichlids. Unfortunately, it is not possible to state which of the fishes collected is the type-host of *N. tanganyikae* [43]. *Neocladocystis congoensis* has been reported from *Parauchenoglanis monkei* (Keilhack, 1910) from Ebogo near the River Nyong in Cameroon [48] and from “an unidentified siluroid fish near Kisangani (“Stanleyville”)” in the Democratic Republic of Congo [70]. Unlike in *N. bemba* n. sp. and the other newly described species *N. biliaris* n. sp. (description follows bellow), in neither species does the vitellarium enter the forebody. A third congeneric species, *N*. *intestinalis* (Vaz, 1932) Manter & Pritchard, 1969, was reported from the South American characiform *Salminus brasiliensis* (Cuvier) in the Paraná River, Argentina, with several fish species as second intermediate hosts [71, 72]. *Neocladocystis bemba* n. sp. is distinguished from *N. congoensis* and *N. tanganyikae* by a combination of characters including the relative position of ovary and seminal receptacle, size of eggs and relative size of the oral sucker, together with the presence of entire ovary and testes and the distribution of the vitelline fields which may extend from about the level of the ventral sucker to the post-testicular region almost to the posterior body extremity.

***Neocladocystis biliaris* Georgieva, Kmentová & Bray n. sp.**

***Type-host*:***Lates mariae* Steindachner (Actinopterygii: Latidae).

***Type-locality*:** Lake Tanganyika at Uvira (4°20ʹS, 29°09ʹE), Democratic Republic of the Congo.

***Type-specimens*:** The holotype (HU 756) and 3 paratypes (HU 757-59) were deposited in the Collection of Hasselt University, Diepenbeek, Belgium.

***Site in host*:** Gall-bladder.

***Representative DNA sequences*:** GenBank accession number: MN705809 (partial *28S* rRNA gene, domains D1-D3).

***ZooBank registration*:** The LSID for the new name *Neocladocystis biliaris* is urn:lsid:zoobank.org:act:59CA866A-727C-4EC5-B1FE-6FBC539D90EA.

***Etymology*:** The specific name *biliaris* is derived from the Latin *vesica biliaris*, meaning gall-bladder, referring to the infection site of this species in the gall-bladder.

**Description**

[Based on 6 specimens; Fig. 1b, Table 4, Additional file 3: Figure S2.] Body irregular oval, flattened. Tegument smooth. Oral sucker spherical, subterminal. Ventral sucker pre-equatorial, spherical, may be completely obscured by eggs, distinctly smaller than oral sucker. Prepharynx very short, not visible in some specimens. Pharynx oval, longer than wide. Oesophagus often not detectable, occasionally short. Intestinal bifurcation just posterior to pharynx. Caeca blind, narrow, reach to level of posterior margin of posterior testis.

Testes 2, slightly lobed, oblique, contiguous or slightly separated, in posterior half of hindbody. Seminal vesicle elongate-oval, naked, entire, dextral, at level of ventral sucker, posterior end obscured by eggs. Gonotyl absent. Genital pore median, immediately anterior to ventral sucker.

Ovary entire, pre-testicular, at distance from anterior testis. Uterus fills much of body from anterior extremity to mid-testicular region, mostly intercaecal. Eggs numerous, noticeably variable, tanned, operculate. Vitellarium follicular, in 2 lateral fields, reaches from just anterior to ventral sucker to close to posterior extremity, overlapping caeca dorsally and ventrally. Seminal receptacle saccular, dorsal, post-ovarian.

Excretory vesicle Y-shaped, arms extending to forebody, widening anteriorly, narrowing posteriorly, mostly obscured by eggs. Excretory pore terminal.

**Remarks**

*Neocladocystis biliaris* n. sp. differs from its congeners by a combination of characters including the larger body length, entire seminal vesicle and the hitherto unique microhabitat exploited in the fish host, i.e. the gall-bladder. As mentioned above, unlike in *N. biliaris* n. sp. and the other newly described species *N. bemba* n. sp., in neither of other species of *Neocladocystis* does the vitellarium enter the forebody. *Neocladocystis biliaris* n. sp. differs from *N. bembae* n. sp. in the larger body length, in the relative length of the post-testicular region (9.2. *vs* 16.2%) and in the site in the host.

***Neocladocystis* sp.**

***Host*:***Lates angustifrons* Boulenger (Actinopterygii: Latidae).

***Locality*:** Lake Tanganyika at Mpulungu (8°46ʹS, 31°07ʹE), Zambia.

***Site in host*:** Intestine.

***Representative DNA sequences*:** GenBank accession number: MN705810 (partial *28S* rRNA gene, domains D1-D3); MN702816 (*cox*1).

**Remarks**

As only a single immature specimen of *Neocladocystis* sp. lacking species-specific characters was obtained, full morphological description was not possible (Additional file 4: Figure S3). Comparative sequence analysis of *28S* rDNA confirmed the distinct status of this specimen as another species of *Neocladocystis* (the electronic voucher of the specimen used for molecular characterisation is provided in Additional file 4: Figure S3).

***Tanganyikatrema* Kmentová, Georgieva & Bray n. g.**

**Diagnosis**

Body elongate, fusiform, widest at level of ventral sucker or mid-body, length:width ratio *c*.1.7–2.7. Tegument armed with minute spines extending to level to posterior margin of posterior testis. Eye-spots lacking. Oral sucker infundibuliform, muscular, lacking circumoral spines, opens terminally. Ventral sucker subspherical, unspecialised. Sucker-width ratio *c*.1–1.7. Forebody occupies 32–62% of body length. Prepharynx of variable length. Pharynx elongate, muscular. Oesophagus indistinguishable. Caeca reach posterior testis. Testes two, tandem, oval, contiguous or slightly overlapping, entire, in posterior hindbody, close to posterior extremity. Cirrus and cirrus-sac absent; seminal vesicle tubulo-saccular, dorso-sinistral to ventral sucker. Genital pore sinistral, in posterior forebody. Gonotyl absent. Ovary pre-testicular, dextral, entire, close to anterior testis. Uterus restricted to hindbody, anterior to mid-level of anterior testis. Seminal receptacle saccular, relatively large, at level of anterior testis and ovary. Eggs numerous, small, elliptical, tanned, operculate. Vitellarium follicular, follicles in two lateral groups between ventral sucker and level of ovary or anterior testis; excretory pore terminal. *Type-species*: *Tanganyikatrema fusiforma* n. sp.

***Zoobank registration*:** The LSID for the new genus *Tanganyikatrema* is urn:lsid:zoobank.org:act:E93C03BE-98D4-490E-BADD-57435C17C242.

***Etymology*:** The genus name is proposed in reference to Lake Tanganyika to honour this biodiversity hotspot and appended to the commonly used ending -*trema*. It is to be treated as feminine.

**Differential diagnosis**

The only digenean genus parasitic in fish hitherto reported from Lake Tanganyika, *Neocladocystis*, differs from *Tanganyikatrema* n. g. in the presence of a rounded oral sucker (*vs* infundibuliform), short prepharynx and oesophagus (*vs* variable in length prepharynx and long oesophagus), and slightly oblique testes (*vs* tandem). *Neocladocystis* includes species parasitic in cichlid and bagrid fishes in Africa and characid fishes in South America. *Tanganyikatrema* n. g. morphologically resembles *Claribula* Overstreet, 1969, a monotypic genus proposed for species parasitic in marine fishes of the families Albulidae and Sphyraenidae off Florida, USA, by the possession of a fusiform body and a cup-shaped oral sucker, but differs in the presence of spined tegument, prepharynx, oesophagus (in mature individuals), intestinal bifurcation anterior to ventral sucker and testes located in the posterior third of the hindbody. *Isocoelium* Ozaki, 1927 is a genus whose representatives parasitise marine uranoscopid fishes. It resembles the new genus in the presence of tegumental spines, oblique to slightly tandem testes and the uterus reaching no further posteriorly than the testicular zone but differs by having a deeply lobed ovary at mid-hindbody and a shorter forebody in relation to body length.

***Tanganyikatrema fusiforma* Kmentová, Georgieva & Bray n. sp.**

***Type-host*:***Lates microlepis* Boulenger (Actinopterygii: Latidae).

***Other host*:***Lates angustifrons* Boulenger (Actinopterygii: Latidae).

***Type-locality*:** Lake Tanganyika at Katukula (8°36ʹS, 31°11ʹE), Zambia.

***Other localities*:** Lake Tanganyika at Mutondwe Island (8°42ʹS, 31°07ʹE) and Mpulungu (8°46ʹS, 31°07ʹE), Zambia.

***Type-specimens*:** The holotype (NHMUK.2019.11.18.12) and one paratype (NHMUK.2019.11.18.13) were deposited in the Helminthological Collection of the Natural History Museum, London, UK, and 5 paratypes (HU 770-74) were deposited in the Collection of Hasselt University, Diepenbeek, Belgium.

***Site in host*:** Intestine.

***Representative DNA sequences*:** GenBank accession numbers: MN705811 (partial *28S* rRNA gene, domains D1-D3); MN702817 (*cox*1).

***ZooBank registration*:** The LSID for the new name *Tanganyikatrema fusiforma* is urn:lsid:zoobank.org:act:BEA5A2AB-7AF3-4075-8292-4EE688155CAE.

***Etymology*:** The specific name is derived from the Latin *fusiformis* meaning fusiform and referring to the body shape: wide in the middle and tapered at the forebody.

**Description**

**Fig. 3:**
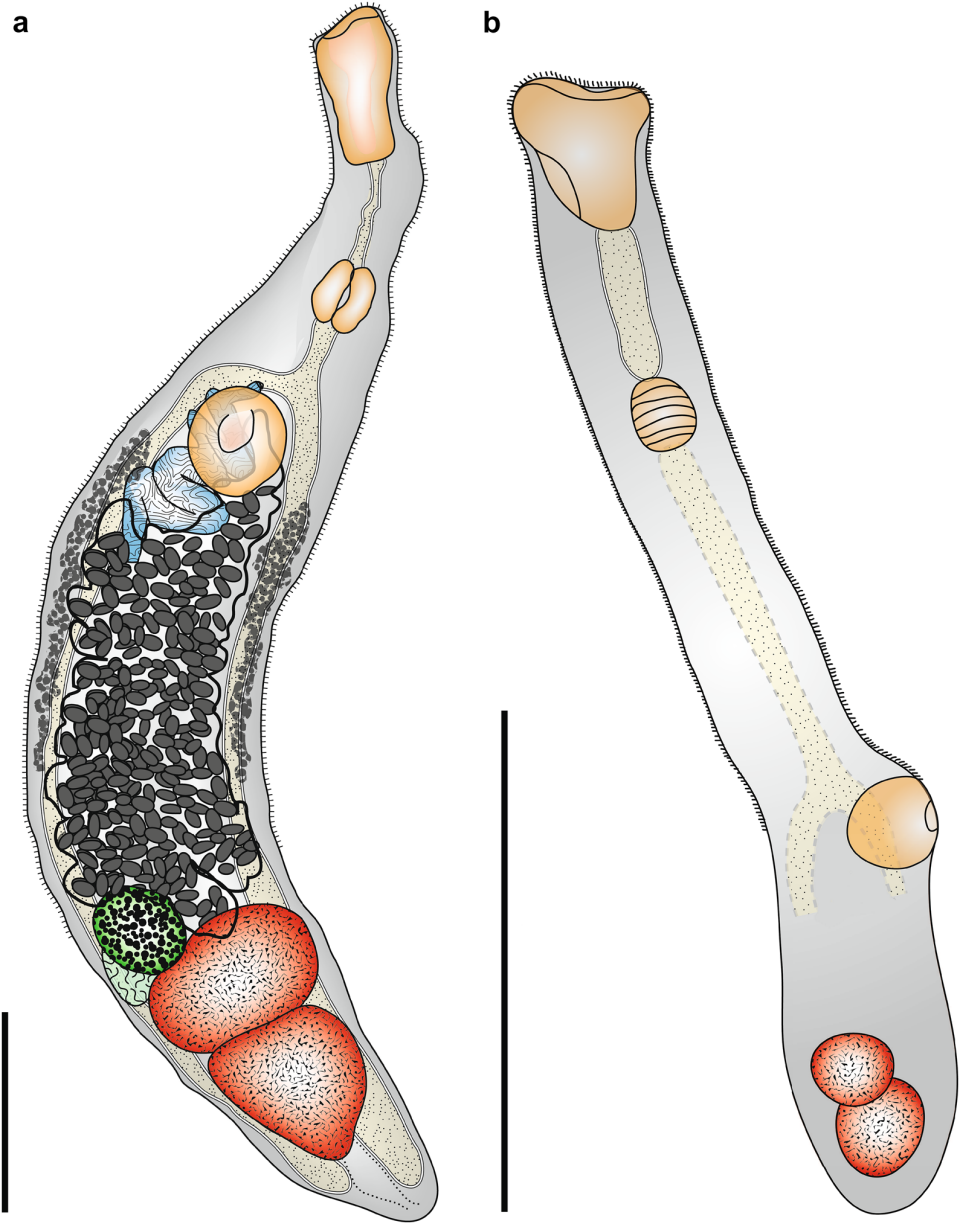
Line drawings of paragenophores of *Tanganyikatrema* spp. **a***Tanganyikatrema fusiforma* n. sp. from the intestine of *Lates microlepis* in Katukula Bay, Lake Tanganyika. **b***Tanganyikatrema* sp. ‛elongataʼ from the intestine of *L. angustifrons* off Mpulungu. *Scale-bars*: 500 µm

[Based on 12 specimens including 3 immature individuals; Fig. 3a, Table 4, Additional file 5: Figure S4.] Body elongate, fusiform, narrow, longer than wide. Tegument spined, spines reach to posterior margin of posterior testis. Eye-spot pigment absent. Oral sucker infundibuliform (distorted in larger worms) or cup-shaped, massive, relatively large, longer than wide, squared-off posteriorly, aperture terminal. Circumoral spines absent. Ventral sucker pre-equatorial, rounded, unspecialised, embedded in ventrogenital sac. Prepharynx long. Pharynx oval, muscular. Oesophagus shorter than prepharynx. Intestinal bifurcation in posterior forebody just anterior to ventral sucker. Caeca end blindly, reach into post-testicular region close to posterior body extremity.

Testes 2, entire, tandem, contiguous or overlapping, in posterior third of hindbody; anterior testis oval, posterior testis subtriangular. Post-testicular region short. Cirrus and cirrus-sac absent. Seminal vesicle tubule-saccular, naked, long, bipartite, convoluted, sinistral to ventral sucker, posterior extent obscured by eggs. No prostatic cells evident. Common genital pore median, immediately antero-sinistral to ventral sucker. Gonotyl absent.

Ovary regularly oval, pre-testicular, anterior or overlapping to anterior testis. Uterus fills much of hindbody from anterior testis anteriorly, passes dorsally to ventral sucker, mostly intercaecal. Mehlis’ gland and Laurer’s canal not observed. Seminal receptacle saccular, contiguous with anterior testis and dorsal to ovary. Eggs numerous, elliptical, malformed in larger worms, operculated, tanned. Vitellarium follicular, in 2 lateral fields, extends from about level anterior to ovary to ventral sucker; laterally overlapping caeca dorsally and ventrally. Seminal receptacle saccular, dorsal, post-ovarian.

Excretory system not clearly visible, pore terminal, vesicle not clearly detected.

***Tanganyikatrema* sp. ‛elongataʼ**

***Host*:***Lates angustifrons* Boulenger (Actinopterygii: Latidae).

***Locality*:** Lake Tanganyika at Mpulungu (8°46ʹS, 31°07ʹE), Zambia.

***Voucher specimens*:** 3 voucher specimens (HU 775-77) were deposited in the Collection of Hasselt University, Diepenbeek, Belgium.

***Site in host*:** Intestine.

***Representative DNA sequences*:** GenBank accession numbers: MN705812 (partial *28S* rRNA gene, domains D1-D3).

***Etymology*:** We distinguish *Tanganyikatrema* sp. ‛elongataʼ from *Tanganyikatrema fusiforma* n. sp. with the epithet ‛elongataʼ derived from the Latin *elongatus* referring to the elongated forebody of the species. This does not intend to be a nomenclatural act and the name should not be interpreted as a species name.

**Description**

[Based on 3 specimens including 2 immature individuals; Fig. 3b, Table 4, Additional file 6: Figure S5.] Body elongate, narrow. Tegument spined, spines scale-like at anterior body extremity diminishing in size posteriorly, extending close to posterior body extremity. Oral sucker infundibuliform, shallow, muscular, aperture terminal, lacking circumoral spines. Ventral sucker pre-equatorial, oval, muscular. Prepharynx long. Pharynx oval, small. Oesophagus, intestinal bifurcation and ending of caeca not clearly visible.

Primordial testes entire, small, tandem, overlapped, in posterior third of hindbody, both testes squared. Seminal vesicle not observed.

Ovary not observed. Uterus and vitelline fields restricted to hindbody. Mehlis’ gland, Laurer’s canal not observed; seminal receptacle not visible.

**Remarks**

The two species of *Tanganyikatrema* n. g. are distinguished from each other by the length:width ratio of the oral sucker (greater in *Tanganyikatrema fusiforma* n. sp.) as well as the forebody:hindbody ratio with the forebody being elongated up to 65% of the body length in *Tanganyikatrema* sp. ‛elongataʼ compared to 41% in immature and 32% in egg-bearing individuals of *Tanganyikatrema fusiforma* n. sp.

***Grandifundilamena* Bray, Kmentová & Georgieva n. g.**

***Diagnosis***

Body elongate, relatively narrow, widest at level of oral sucker, length:width ratio 10.4–11.6. Tegument unarmed. Eye-spots absent. Oral sucker broadly infundibuliform, lacking circumoral spines, opens terminally. Ventral sucker subspherical, in anterior quarter of body, distinctly smaller than oral sucker. Sucker width ratio 2.5–2.9. Forebody occupies 23–24% of body length. Prepharynx relatively short. Pharynx oval, relatively large. Oesophagus short or indistinguishable; intestinal bifurcation in posterior forebody. Caeca reach posterior margin of posterior testis. Testes nine, in tandem series in posterior third of body, reaching close to posterior extremity. Cirrus and cirrus-sac absent. Seminal vesicle long, narrow, tubular, mainly in hindbody. Genital pore immediately anterior to ventral sucker. Gonotyl absent. Vitellarium follicular, in two lateral fields from posterior level of seminal vesicle to level of posterior testis. Ovary lobed, in posterior third of hindbody. Seminal receptacle saccular, in ovarian region. Uterus mainly in hindbody, pretesticular. Excretory pore terminal, vesicle not detected. *Type-species*: *Grandifundilamena novemtestes* n. sp.

***Zoobank registration*:** The LSID for the new genus *Grandifundilamena* is urn:lsid:zoobank.org:act:7DCCF6E3-3115-45F0-A3BF-D6DC04325342.

***Etymology*:** The genus name is derived from the Latin *grandis* meaning grand and combination of *infundibuli* and *vitulamena* referring to the funnel shape of oral sucker. It is to be treated as feminine.

**Differential diagnosis**

*Grandifundilamena* n. g. is distinguished from the other cryptogonimid genera by the combination of an infundibuliform oral sucker, the pharynx being larger than the ventral sucker, the vitelline fields extending from about mid-hindbody to nearly the posterior body extremity, the entire ovary, the seminal vesicle anterior to the ovary and the possession of nine tandem testes. Several cryptogonimids are reported to possess multiple testes, e.g. representatives of *Polyorchitrema* Srivastava, 1939 (infecting members of the Sparidae), *Iheringtrema* Travassos, 1947 (infecting members of the Pimelodidae), *Siphodera* (infecting members of many families, primarily the Lutjanidae), *Siphomutabilis* Miller & Cribb, 2013 (infecting members of the Lutjanidae), *Novemtestis* Yamaguti, 1942 (metacercariae in members of the Mullidae, host of adults unknown), *Acanthosiphodera* Madhavi, 1976 (infecting members of the Lutjanidae). The common difference between *Grandifundilamena* n. g. and species of *Polyorchitrema*, *Iheringtrema*, *Siphodera*, *Siphomutabilis* as well as *Acanthosiphodera* lies in the presence of an infundibuliform oral sucker (*vs* round and a larger body length:width ratio). Additionally, the position of vitelline fields restricted to the hindbody and the absence of oral spines distinguish *Grandifundilamena* n. g. from species of *Novemtestis*. *Grandifundilamena* n. g. resembles *Mitotrema anthostomatum* Manter, 1963, a species infecting serranid fishes in the Pacific Ocean, in the presence of an infundibuliform sucker combined with elongated body but differs in the relative length of the forebody (23–24 *vs* 10%) and the number of testes (9 *vs* 2). Based on molecular evidence, Miller & Cribb [73] have shown that closely related species may have two or multiple testes. Thus, the type-species of *Siphomutabilis*, *Siphomutabilus gurukun* (Machida, 1986) Miller & Cribb, 2013, possesses nine testes arranged in a longitudinal row, as observed in *Grandifundilamena* n. g.; *Siphomutabilus aegyptensis* (Hassanine & Gibson, 2005) Miller & Cribb, 2013 [74] has been reported with nine testes distributed as in a ring, and both *S. raritas* Miller & Cribb, 2013 and *S. bitesticulatus* Miller & Cribb, 2013 have been reported having two testes [73–75].

***Grandifundilamena novemtestes* Bray, Kmentová & Georgieva n. sp.**

***Type-host*:***Lates angustifrons* Boulenger (Actinopterygii: Latidae).

***Type-locality*:** Lake Tanganyika at Mpulungu (8°46ʹS, 31°07ʹE), Zambia.

***Type-specimens*:** The holotype (NHMUK.2019.11.18.10) and one paratype (NHMUK.2019.11.18.11) were deposited in the Helminthological Collection of the Natural History Museum, London, UK, and one paratype (HU 778) was deposited in the Collection of Hasselt University, Diepenbeek, Belgium.

***Site in host*:** Intestine.

***ZooBank registration*:** The LSID for the new name *Grandifundilamena novemtestes* is urn:lsid:zoobank.org:act:65F6172F-BA12-4888-A2EA-6B7D30C6A3E3.

***Etymology*:** The specific name is derived from the Latin as a combination of *novem* and *testes*, referring to the nine testes present in *Grandifundilamena novemtestes* n. sp.

**Description**

**Fig. 4:**
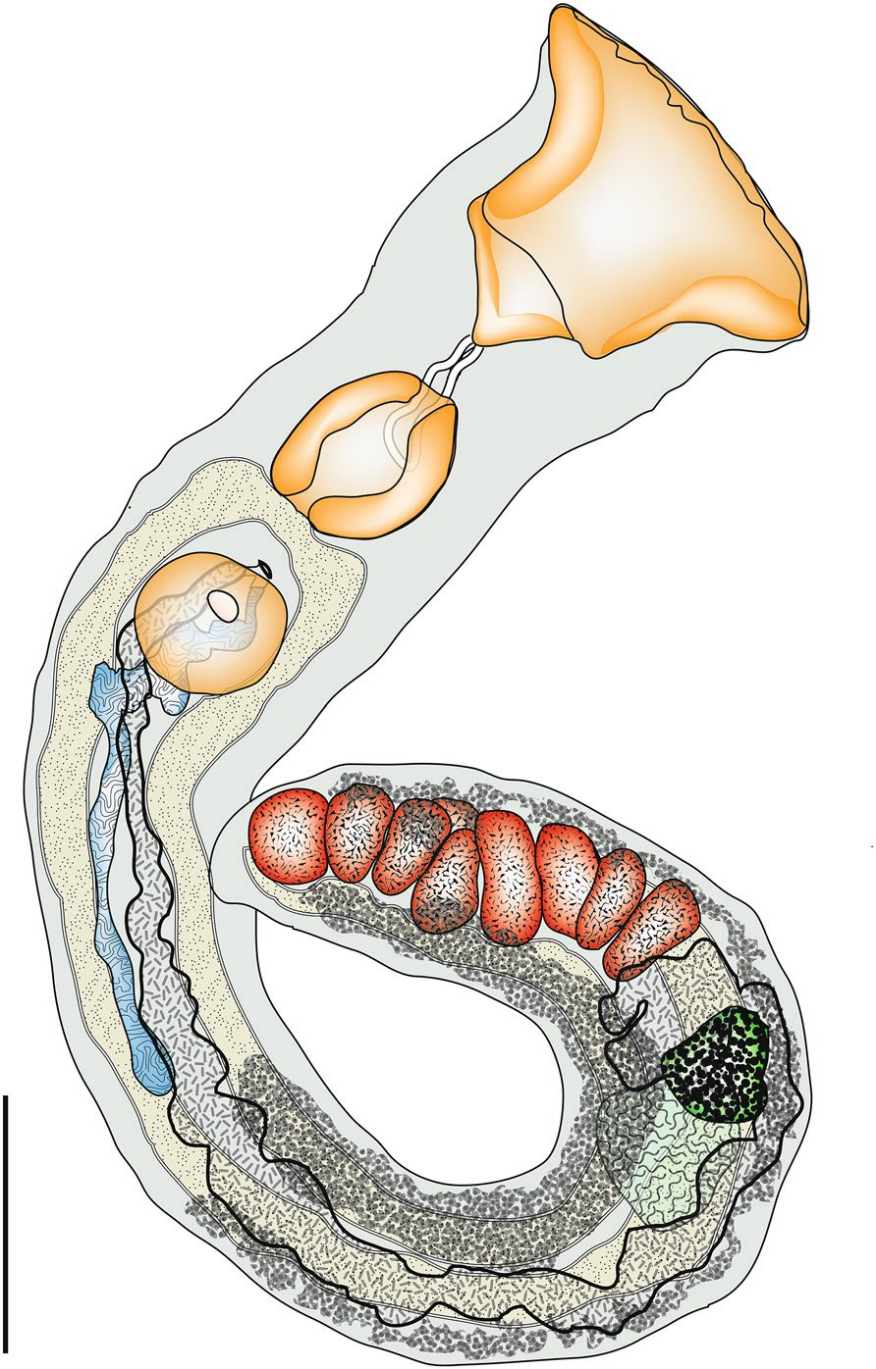
Line drawing of *Grandifundilamena novemtestes* n. sp. Specimen recovered in the intestine of *Lates angustifrons*, Mpulungu fish market, Lake Tanganyika. *Scale-bar*: 500 µm

[Based on 3 specimens; Fig. 4, Table 4, Additional file 7: Figure S6.] Body long, relatively narrow, widest at oral sucker, body widest in anterior forebody (for width measurements), tapering gradually, tends to take up curved position on slides and is difficult to mount fully dorso-ventrally. Tegumental spines not detected. Oral sucker massive, broadly infundibuliform may extend as triangle posteriorly, aperture wide, terminal. Ventral sucker pre-equatorial, rounded, much smaller than oral sucker. Prepharynx short, narrow. Pharynx broadly oval, larger than ventral sucker. Oesophagus absent. Intestinal bifurcation in posterior forebody. Caeca wide, end blindly, reach into post-testicular region close to posterior body extremity.

Testes 9, transversely-oval, entire, small, in tandem row, reaching from just posterior to seminal receptacle to close to posterior body extremity; contiguous, in posterior third of hindbody. Seminal vesicle naked, long, convoluted, extended posteriorly, obscured by eggs. Genital pore median, immediately anterior to ventral sucker.

Ovary irregularly subtriangular, pretesticular, at some distance from anterior testis. Seminal receptacle saccular, anterior to ovary. Uterus narrow, between ventral sucker and anterior testis, passes dorsally to ventral sucker, mostly intercaecal. Eggs small, tanned, operculate. Vitellarium follicular, fields reach from level of posterior margin of seminal vesicle to about level of posterior testis, post-vitelline field short.

Excretory pore terminal, vesicle not traced beyond posterior testis.

Detailed morphological comparative data of the newly described genera and already described morphologically similar cryptogonimid genera are provided in Table 5.

**Table 5:** Comparative data for morphological characters among the selected cryptogonimid genera based on [44] and this study

	*Neocladocystis* Manter & Pritchard, 1969 (amended diagnosis)	*Tanganyikatrema* n. g.	*Grandifundilamena novemtestes* n. g., n. sp.	*Acanthostomum* Looss, 1899	*Brientrema* Dollfus, 1950	*Claribulla* Overstreet, 1969	*Iheringtrema* Travassos, 1947	*Isocoelium* Ozaki, 1927	*Siphodera* Linton, 1910
Body shape	Irregular oval	Elongate, fusiform	Long, relatively narrow	Elongate-oval to distinctly elongate	Fusiform	Elongate with distinct constriction immediately posterior to oral sucker	Elongate-oval	Very elongate	Oval, enlongate-oval
BL/BW	1.5–2.5	4–7	*c.*11	2–3.5	2–2.5	*c.*6	*c.*3	11–16	2–3.5
OS	Almost round, opens subterminally	Infindibular or cup-shaped, massive, relatively large, longer than wide, squared-off posteriorly, opens terminally	Massive, broadly infundibular, opens terminally	Funnel-shaped, opens terminally	Nearly round, opens terminally	Funnel- or cup-shaped, opens almost terminally	Almost round, opens subterminally	Longer than wide, opens subterminally	Nearly round, opens almost terminally
Circumoral spines	Absent	Absent	Absent	Enlarged (rarely absent)	Enlarged	Absent	Absent	Absent	Absent
VS	Pre-equatorial, rounded, unspecialised, not obviously embedded in ventrogenital sac	Pre-equatorial, rounded, unspecialised, embedded in ventrogenital sac	Pre-equatorial, rounded, much smaller than oral sucker.	Pre-equatorial, rounded, unspecialised, not obviously embedded in ventrogenital sac	Pre-equatorial, rounded, unspecialised, embedded in ventrogenital sac	Pre-equatorial, rounded unspecialised, deeply embedded in ventrogenital sac	Pre-equatorial, rounded, unspecialised, not obviously embedded in ventrogenital sac	Pre-equatorial, rounded, unspecialised, not obviously embedded in ventrogenital sac	Pre-equatorial, rounded unspecialised, embedded in ventrogenital sac, usually smaller than VS
OSW/VSW	1.5–2.1	0.8–1.7	*c.*2.7	1–2.5	1.5–2.5	*c.*2.5	2.0–2.5	*c.*2.0	1–2.5
Tegument	Smooth or spined, spines reach to posterior extremity, largest in mid-region	Spined, spines can reach at about level of ovary	Spines not observed	Smooth or spined, spines reach to posterior extremity	Spined, spines reach to posterior extremity	Spines not observed	Spines not observed	Spines not observed	Spined
Forebody	26–40% of BL	30–62% of BL	*c.*24 % of BL	5–40% of BL	20–25% of BL	*c.*40% of BL	*c*.20% of BL	20–25% of BL	20–33% of BL
Prepharynx	Short or absent	Variable in length	Broadly oval, larger than ventral sucker	Short	Very short	Very short	Very short	Short	Short
Oesophagus	Short or indistinct	Shorter than prepharynx	Absent	Short	Very short	Short	Very short	Long	Short
Intestinal bifurcation	In about mid-forebody, at level of ventral sucker	In posterior forebody just anterior to VS	In posterior forebody	Immediately anterior to VS	Immediately anterior to or dorsal to VS	In anterior forebody	In mid-forebody	In posterior forebody	In mid-forebody
Caeca	Blind, end at level of testes or post-testicular region	Blind, reach into post-testicular region	Blind, reach into post-testicular region	Opens *via* separate ani close to posterior extremity	Blind, end close to posterior extremity	Blind, end posteriorly to testes	Blind, end close to posterior extremity	Blind, end close to posterior extremity	Blind, end close to posterior extremity
Testes	2, symmetrical or oblique, lobed, contiguous or slightly separated	2, entire, tandem, contiguous, in posterior third of hindbody; anterior testis oval, posterior testis sub-triangular	9, transversely oval, entire, in tandem row, contiguous, in posterior third of hindbody	2, entire, tandem, contiguous, in posterior third of hindbody	2, symmetrical to slightly oblique, close to posterior extremity	2, strongly oblique to tandem, in mid-hindbody	9, irregular, in posterior hindbody	2, mostly intercaecal, distinctly elongate, strongly oblique to slightly tandem	Usually 9, in two lateral groups, or one large group in hindbody
Seminal vesicle	Elongate-oval, naked	Tubulo-saccular, naked, long, convoluted	Naked, long, convoluted	Tubular	Tubulo-saccular	Tubulo-saccular, in forebody	Tubular	Tubular	Tubulo-saccular
Gonotyl	Absent	Absent	Absent	Absent	A spined muscular structure immediately anterior to VS	Absent	Absent	Absent	Absent
Genital pore	Median, immediately anterior to VS	Median, immediately anterior to VS	Median, immediately anterior to VS	Median, immediately anterior to VS	Median, immediately anterior to VS	Median, at mid-level of intestinal bifurcation and VS	Sinistral to VS	Median, immediately posterior to VS	Median, posterior and slightly sininstral to VS
Ovary	Dextral, pretesticular, regularly lobed	Oval, pre-testicular, overlapping anterior testis	Irregularly subtriangular, pre-testicular	Entire, pre-testicular; in posterior hindbody	Entire, anterior to testes in posterior hindbody	Entire, immediately anterosinistral to anterior testis	Deeply lobed, median, immediately anterior to testes	Deeply lobed, well separated form anterior testis, occupies full width of body in mid-hindbody	Deeply lobed, immediately anterior to testes
Uterus	Fills much of body from intestinal bifurcation to anterior testicular region, mostly intercaecal	Fills much of hindbody from anterior testis anteriorly, passes dorsally to VS, mostly intercaecal	Narrow, reaches between VS and anterior testis, passes dorsally to VS, mostly intercaecal	In hindbody between ovary and VS	In hindbody between ovary and VS	In hindbody, extends close to posterior extremity	In hindbody, between gonads and VS	In hindbody, between VS and anterior testis	In hindbody, extends close to posterior extremity
Vitelline follicles	In 2 lateral groups from just post intestinal bifurcation to close to posterior extremity, just overlapping caeca	In 2 lateral groups, reach from VS to level distinctly anterior to ovary, just overlapping caeca dorsally and ventrally	In 2 lateral groups, reach from about halfway between VS and ovary to level of posterior testis	In 2 lateral groups, in hindbody, from slightly posterior to VS to level of anterior testis or mid-body	In 2 lateral groups, in hindbody, from slightly anterior to ovary to the posterior extremity	In 2 restricted lateral groups between VS and gonads	In 2 lateral groups, confluent posteriorly, from level of pharynx to posterior extremity	In 2 bands, occupy full body width from midway between VS to ovary and ovary to anterior testis	In 2 lateral groups, may extend from forebody to level of testes
Excretory vesicle	Y-shaped, bifurcates lateral to ovary, narrow posteriorly, widens and reaches uterus	Not clear	Not clear	Y-shaped, bifurcated	Bifurcated	Bifurcated	Unknown	Bifurcated	Y-shaped, bifurcated
Excretory vesicle arms	May extend to level of pharynx, excretory pore terminal at posterior end of body	Not clear	Not traced beyond posterior testis	Reach level of pharynx	Reach pharynx	Reach pharynx	Unknown	Reach pharynx	Reach level of pharynx
Hosts	Freshwater fishes (Latidae: *L*. *angustifrons*, *L. mariae*, *L*. *microlepis*)	Freshwater fishes (Latidae: *L*. *angustifrons*, *L*. *microlepis*)	Freshwater fishes (Latidae: *L*. *angustifrons*, *L*. *microlepis*)	Freshwater and marine fishes; reptiles	Freshwater fishes (Malapteruridae, Citharinidae and pelicans - probable pseudoparasitism)	Marine fishes (Albulidae, Sphyraenidae)	Freshwater fishes (Pimelodidae)	Marine fishes (Uranoscopidae)	Marine fishes
Site in host	Immature specimens in intestine; eggs-bearing specimens in pyloric caeca and gall-bladder	Intestine	Intestine	Intestine	Intestine	Intestine	Intestine	Intestine	Intestine
Distribution	Africa (Lake Tanganyika)	Africa (Lake Tanganyika)	Africa (Lake Tanganyika)	Cosmopolitan	Africa	North America (Florida, USA)	South America (Brazil)	Japan	Atlantic, Indian and Pacific Oceans
Type-species	*N*. *tanganyikae* (Prudhoe, 1951) Manter & Pritchard, 1969	*Tanganyikatrema fusiforma* n. g., n. sp.	*Grandifundilamena novemtestes* n. g., n. sp.	*A. spiniceps* (Looss, 1896) Looss, 1899	*B*. *pelecani* Dollfus, 1950	*C*. *longula* Overstreet, 1969	*I. iheringi* Travassos, 1947	*I. mediolecithale* Ozaki, 1927	*S. vinaledwardsii* (Linton, 1901) Linton, 1910

*Abbreviations*: BL, body length; BW, body width; OS, oral sucker; OSW, oral sucker width; VSW, ventral sucker width; VS, ventral sucker

**Molecular characterisation and phylogeny**

The newly obtained sequences of the *28S* rDNA region (1240 bp) represented five distinct genotypes, which correspond to morphologically distinct species recovered in this study, confirming the presence of five species. Interspecific sequence divergence ranged between 1–25 bp (0.1–2.1%) (see Table 6 for further details). Replicate specimens of the two most abundant genotypes of *N. bemba* n. sp. (5 specimens ex *L*. *microlepis* and 2 specimens ex *L. angustifrons*) and *T. fusiforma* n. sp. (3 specimens ex *L*. *microlepis* and 2 specimens ex *L. angustifrons*) shared identical *28S* rDNA sequences. A single sequence of *N. biliaris* n. sp. recovered from *L*. *mariae* at Uvira differed by a single nucleotide from *N. bemba* n. sp. An individual of *Tanganyikatrema* sp. ‘elongata’ that was genetically characterised differed from *T. fusiforma* n. sp. by four nucleotides in the *28S* rDNA gene portion. Representative single genotypes per species were used in the phylogenetic analyses. Two of the genotypes were shared between parasites of *L*. *microlepis* and *L. angustifrons* (i.e. represented by 7 and 5 isolates, respectively) while the remaining three genotypes represented unique sequences from specimens recovered from three of the host species, i.e. *L*. *mariae*, *L*. *microlepis* and *L*. *angustifrons*.

**Table 6:** Total pairwise differences among partial *28S* rDNA sequences for the cryptogonimid species reported in this study

Species		1	2	3	4	5
1	*Neocladocystis bemba* n. sp.	–	0.1	0.5	1.9	1.9
2	*Neocladocystis biliaris* n. sp.	1	–	0.6	2.0	2.0
3	*Neocladocystis* sp.	6	7	–	1.6	1.8
4	*Tanganyikatrema fusiforma* n. sp.	24	25	20	–	0.3
5	*Tanganyikatrema* sp. ‛elongataʼ	24	25	22	4	–

*Note*: Uncorrected pairwise differences (below the diagonal) and mean divergence (uncorrected p-distance in % above the diagonal) among the newly discovered cryptogonimid species from *Lates* spp. in Lake Tanganyika

Partial *cox*1 sequences were obtained for 9 isolates from 3 of the 6 cryptogonimid trematode species, i.e. 7 *Neocladocystis bemba* n. sp. ex *L*. *microlepis* and a single sequence each for *Neocladocystis* sp. and *T. fusiforma* n. sp., both recovered from *L*. *microlepis*. Comparative sequence analysis revealed high levels of intra- and interspecific genetic divergence. The intraspecific genetic divergence for the isolates of *N*. *bemba* n. sp. ranged between 1.3–4.2% (10–33 bp difference). All isolates of *N*. *bemba* n. sp. represented unique haplotypes. Sequence divergence between *N*. *bemba* n. sp. and *Neocladocystis* sp. ranged between 15.5–16.2% (123–129 bp) divergence and 17.1–18.5% (136–147 bp) difference between *N*. *bemba* n. sp. and *T. fusiforma* n. sp. Further, the sequences of *Neocladocystis* sp. and *T. fusiforma* n. sp. differed considerably, i.e. by 19.4% (154 bp) difference.

**Fig. 5:**
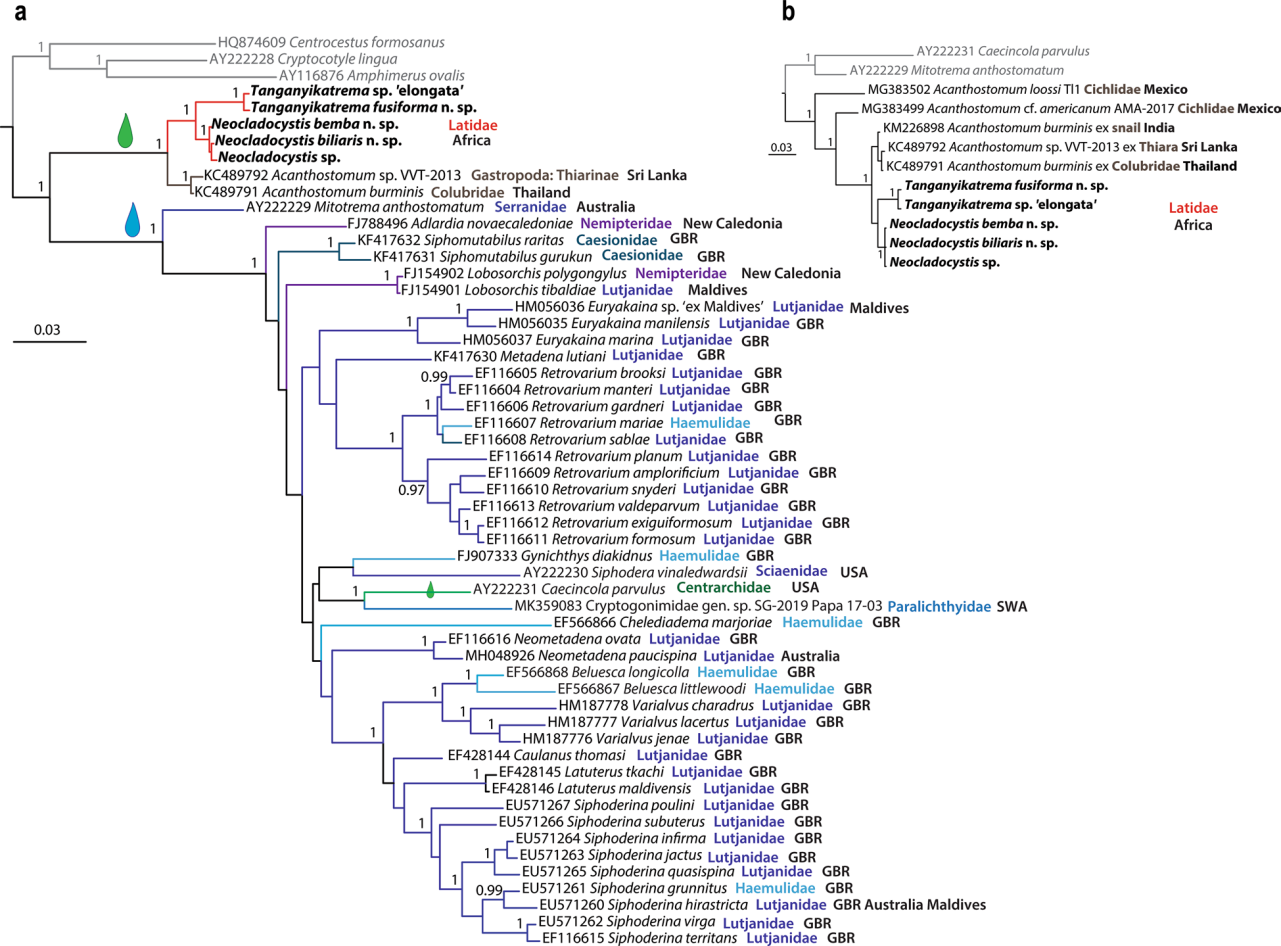
Bayesian inference phylogram based on partial *28S* rDNA sequences (domains D1-D3) for the Cryptogonimidae (**a**) and *Acanthostomum* spp. and the newly sequenced cryptogonimid representatives from Lake Tanganyika (**b**). Both phylograms were inferred under the GTR+Г model of nucleotide substitution. Only posterior probability values > 0.95 are shown. The new cryptogonimid species described here are highlighted in bold. Freshwater and marine origin of the species is indicated by a green and a blue drop, respectively. Host family specification and place of origin of the ingroup taxa are indicated. The scale-bar represents the number of nucleotide substitutions per site. *Abbreviations*: GBR, Great Barrier Reef; SWA, South-West Asia

Phylogenetic relationships among representatives of the Cryptogonimidae were assessed based on a dataset including 52 taxa (Fig. 5a). Overall, they clustered into two major clades, i.e. (i) one formed by the freshwater representatives of *Acanthostomum*; and (ii) a second major clade including the remaining currently available sequences for cryptogonimids, all reported from marine fishes except for *Caecincola parvulus* Marchal & Gilbert, 1905 which was recovered from the freshwater centrarchid *Micropterus salmoides* (Lacépède) in the USA. *Mitotrema anthostomatum* Manter, 1963 diverged earlier among the marine cryptogonimids. Despite the large number of sequences available for marine cryptogonimids, BI analysis did not lend much statistical support for the major nodes and indicated a lack of phylogenetic resolution. The newly obtained sequences from Lake Tanganyika clustered with species of *Acanthostomum* reported from Asia in a strongly supported clade.

Relationships among the newly sequenced isolates from Lake Tanganyika were further assessed based on a restricted dataset including only the currently available isolates of *Acanthostomum* (Fig. 5b). The novel isolates from Tanganyika formed a strongly supported clade sister to a clade comprising sequences for *Acanthostomumburminis* (Bhalerao, 1926) Bhalerao, 1936 from India and Thailand and an unidentified digenean labelled as *Acanthostomum* sp. VVT-2013 ex the gastropod *Mieniplotia scabra* (Müller). The African isolates from *Lates* spp. formed two strongly supported sister clades: (i) a clade comprised by species of *Tanganyikatrema* n. g.; and (ii) a clade consisting of *Neocladocystis* spp. The remaining two isolates for *Acanthostomum* cf. *americanum* (Vigueras, 1957) Herber, 1961 and *A*. *loossi* (Vigueras, 1957) Groschaft & Barus, 1970 clustered as basal to the clade of *A*. *burminis* plus the novel isolates from Lake Tanganyika.

## Discussion

The present study provides the first estimates of the trematode diversity in lates perches in Lake Tanganyika. Employing morphological characterisation and phylogenetic inference based on sequence data for the *28S* rRNA gene, the presence of six cryptogonimid species parasitic in three of the four *Lates* spp. endemic to Lake Tanganyika was revealed. All of the recovered cryptogonimid trematodes represent species new to science. The presence of *Neocladocystis* in the lake, first reported by Prudhoe [43], was confirmed, with three new species being recovered. The unique morphological characters of three further species described in the present study and their phylogenetic distinctiveness required the erection of two new genera.

### Cryptogonimid trematodes in Africa

All specimens in the present study possessed morphological characters typical for cryptogonimid digeneans: testes at distance from posterior extremity, extensive uterus, gonotyl absent, common genital pore opening just anterior to ventral sucker, a Y-shaped excretory vesicle, tanned eggs and a lack of cirrus and cirrus-sac. In total, six cryptogonimid species of three genera are described from three latid hosts, including the erection of two new genera. Although Lake Tanganyika has been studied for several decades, the present study is the first to provide molecular data for digenean trematodes in this biodiversity hotspot. Furthermore, only three species of the Latidae, i.e. *L*. *niloticus*, *L*. *calcarifer* and *Psammoperca waigiensis* (Cuvier) have been previously screened for endohelminth parasites (see Table 1 and references therein). Therefore, our study significantly increases the knowledge on the parasite fauna in lates perches, an economically important group for fisheries worldwide.

Currently, only seven cryptogonimid species of four genera, *Acanthostomum*, *Brientrema*, *Neocladocystis* and *Siphodera*, have been reported from African freshwater fishes [43, 46–48]. Of these, two species [*Acanthostomum absconditum* (Looss, 1901) Gohar, 1934 and *A*. *spiniceps* (Looss, 1896) Looss, 1899] were recorded from a bagrid fish host, two were recorded from claroteids (*Neocladocystis congoensis* and *Siphodera ghanensis* Fischthal & Thomas, 1968) and a single species each were recorded from a gymnorchiid [*A*. *gymnarchi* (Dollfus, 1950) Yamaguti, 1958], a malapterurid (*Brientrema malapteruri* Dollfus, 1950) and unidentified fish hosts (*Neocladocystis tanganyikae*), respectively [25, 26, 43, 45–49].

To date, only three species of *Neocladocystis* are known worldwide, of which two, *N. congoensis* and *N. tanganyikae*, were described from African freshwater fishes [43, 48]. Interspecific variability is seen mainly in the mutual position of the bifurcation of the oesophagus and the ventral sucker and in the extent of the vitelline follicles and the sucker ratios [43, 48, 72]. Combined morphological and molecular characterisation of the cryptogonimids recovered in Lake Tanganyika allowed us to assign three of them to *Neocladocystis*. Interestingly, intraspecific phenotypic variability of *N. bemba* n. sp. was combined with a high morphological homogeneity across the three new species of *Neocladocystis*, indicating the presence of a species complex and recent speciation events. In the present case, host species and geographical origin as well as localisation in the host could be the driving force for the divergence between *N. bemba* n. sp. and *N. biliaris* n. sp. This is comparable to the evolution of other cryptogonimids, *Retrovarium formosum* Miller & Cribb, 2007 and *Retrovarium exiguiformosum* Miller & Cribb, 2007, which both infect the chinamanfish, *Symphorus nematophorus* (Bleeker) (Lutjanidae: Perciformes), and were reported from distant geographical areas in the Great Barrier Reef [76]. A similar evidence for high levels of morphological homogeneity was previously documented for the species of *Euryakaina* Miller, Adlard, Bray, Justine & Cribb, 2010 (Cryptogonimidae) [77], though these species are distinguished by notably larger distances in the *28S* rDNA as compared to the difference detected between *N. bemba* n. sp. and *N. biliaris* n. sp. Unfortunately, as only a single immature individual of the third putative new species of *Neocladocystis*, i.e. *Neocladocystis* sp., collected from *L. angustifrons* was available, this did not allow us to provide a full species description. However, its distinct species status was confirmed by a difference of six and seven bp in the *28S* rDNA sequences compared with *N. bemba* n. sp. and *N. biliaris* n. sp., respectively.

Although the cryptogonimids typically have a three-host life-cycle with adults that are localised in the intestine or pyloric caeca [44], adult specimens of *N. biliaris* n. sp. were localised in the gall-bladder of *L. mariae*. Therefore, a potential localisation outside the digestive tract, more specifically in the gall-bladder, was added to the generic diagnosis.

A difference of four bp in the *28S* rRNA gene was found between the two species of *Tanganyikatrema* n. g. Morphologically, the two species mainly differed in the relative position of the ventral sucker. Unlike in the case of *N. bemba* n. sp. and *N. biliaris* n. sp., the two species of *Tanganyikatrema* n. g. were collected from the same host species and locality.

*Grandifundilamena novemtestes* n. g., n. sp. possessed unique morphological characters not only among the cryptogonimids discovered in the present study but also among all currently known cryptogonimid trematodes. The presence of multiple testes, a character rarely seen not only among the cryptogonimids [44] but also among the digenetic trematodes in general, and the possession of a wide, strongly muscular and infundibular oral sucker support the erection of the new genus. Unfortunately, the limited number of specimens collected prevented us from conducting molecular characterisation and phylogenetic placement of *G. novemtestes* n. sp.

There are more endohelminth species yet to be discovered in the largely unexplored fish fauna in Lake Tanganyika. Collecting novel material from distinct localities along the lake would reveal the real magnitude of the trematode species diversity in the lates perches. Additional material is needed to reveal their actual geographical distribution in the lake. Further, clarification of host species of *N. tanganyikae* along with generating sequence data for this species is needed to improve the generic diagnosis.

### Biodiversity in Lake Tanganyika’s pelagic zone

The biodiversity in Lake Tanganyika is concentrated mainly in the littoral zone, which offers unique opportunities for within-lake diversification currently documented for a number of vertebrate and invertebrate species such as cichlid fishes [78], crustaceans [79], poriferans [80] and gastropods [3, 81]. Cryptogonimid digeneans are known to parasitise gastropod invertebrates as first intermediate hosts and fishes as second and definitive hosts. The metacercarial stage is trophically transmitted to the definitive host. Considering that trematode parasites are largely dependent on the local food web and the species interactions involved, the species reported here could therefore provide a link between the highly biodiverse littoral lake zone and the wide pelagic habitat. Unfortunately, so far, there have been no studies on the larval trematode diversity in the lake. The lake’s pelagic zone is inhabited by less diverse fish assemblages including lates perches, clupeids and representatives of some cichlid tribes [16, 20, 21, 23, 82]. In general, parasites recovered from meso- and/or bathypelagic freshwater and marine hosts tend to show low host specificity and limited diversity [11, 16, 83–85]. However, as the overall endohelminth biodiversity in the lake’s littoral habitat is unknown, it remains unclear how it compares to the digenean diversity in the pelagic zone.

Despite the relatively large number of examined fish individuals, neither digenean nor monogenean, cestode or acanthocephalan parasites [16] were recovered from *L. stappersii* so far. This might be related to the different life history and diet preferences compared to its congeners. Whereas as juveniles of *L. mariae* and *L. microlepis* are found in the shallow littoral zone, as adults, they are exclusively pelagic top predators. They differ in their preferred depth of occurrence, with *L. mariae* typically found at greater depths [17, 86]. *Lates angustifrons* is characterised by its preference for specific inshore rocky habitat and a predominantly solitary and more sedentary lifestyle compared to the above-mentioned species. Unlike other congeners in the lake, *L. stappersii* exhibits a truly pelagic lifestyle forming large groups that prey upon clupeids [17, 87]. Consequently, for *L. stappersii* closer contact and/or habitat sharing with gastropods as intermediate hosts for the trematode parasites is rather limited [82].

### Phylogenetic relationships

The phylogeny of the Cryptogonimidae has been the subject of a number of studies with more extensive surveys conducted in the Indo-Pacific region at the Great Barrier Reef (see [76, 88, 89] and reference therein). Despite the limited sequence data available for freshwater cryptogonimid species, our study demonstrates that freshwater parasitism within the family occurs in at least two independent lineages (see Fig. 5a). Apart from the above mentioned earlier diverging clade of *Acanthostomum* spp. including the novel lineage from Lake Tanganyika, *Caecincola parvulus* Marshall & Gilbert, 1905 reported from the freshwater centrarchid *Micropterus salmoides* in the USA, is the only other freshwater cryptogonimid, but clustered within the major marine clade. Further, the monophyly of species parasitic in caesionid, haemulid, lutjanid and nemipterid fish hosts was rejected, possibly indicating multiple switching events between major definitive host groups through their evolution.

The analysis based on the *28S* rDNA sequences confirmed the distinct status of both genera for which sequence data were obtained. In this respect, the recognition of *Neocladocystis* as a distinct genus and the erection of *Tanganyikatrema* n. g. are justified based on both morphological and molecular evidence. Contrasting patterns of diversification have been revealed in the three new genera described here. The diversification events were associated with morphological divergence indicating that similar environmental/microhabitat contexts do not always imply similar outcomes of diversification [90]. Similarly, despite the striking morphological differences among the genera found in Lake Tanganyika’s lates perches, the phylogenetic analyses showed that two of them form a strongly supported clade sister to *Acanthostomum* (Fig. 5a). This further highlights the importance of taxon-dependent factors for the processes involved in their diversification. Three representatives of *Acanthostomum* are known to infect a wide range of fish species as definitive hosts. These include members of distinct families such as the Bagridae, Gymnarchidae and Latidae in Africa, with *L. niloticus* reported as a host of two species of *Acanthostomum* in Egypt [25, 56]. Recent diversification processes within this digenean lineage, indicated by subtle differentiation between the reported congeners both at morphological and DNA sequence level, correspond with the assumed recent invasion and subsequent diversification of the lates perches in Lake Tanganyika ([16] and own unpublished data). However, the lack of parasitological data, especially for endohelminths, in the lake prevents any further conclusions regarding the host specificity of this digenean lineage.

## Conclusions

Six cryptogonimid trematode species belonging to three genera were reported from the lates perch hosts endemic to Lake Tanganyika. Substantial intraspecific phenotypic variability combined with interspecific morphological similarity and contrasting with clear genetic differentiation has been recognised in the recovered species of *Neocladocystis*. Therefore, recent speciation driven by host species preference and/or geographically dependent diversification is hypothesised. Future investigations based on additional material and more and/or faster evolving molecular markers is needed to assess the real levels of intraspecific variation in the cryptogonimid trematodes from Lake Tanganyika. The novel molecular data gathered here indicate the existence of an exclusively freshwater clade within the cryptogonimid genera. The present results highlight the importance of concerted efforts and application of an integrative approach to the assessment of the real biodiversity in this unique ecosystem.

## Supplementary information

**Additional file 1: Table S1.** Summary data for *28S* rDNA sequences retrieved from the GenBank database for species used in the phylogenetic analyses.

**Additional file 2: Figure S1.** Photomicrographs of a paragenophore specimen of *Neocladocystis bemba* n. sp. **a** Body, ventral view. **b** Anterior body extremity with oral sucker. **c** Mid-body. **d** Ovarian and testicular region. **e** Posterior part of hindbody. *Scale-bars*: 100 µm.

**Additional file 3: Figure S2.** Photomicrographs of a paragenophore specimen of *Neocladocystis biliaris* n. sp. **a** Body, ventral view. **b** Anterior body extremity with oral sucker. **c** Mid-body. **d** Ovarian and testicular region. **e** Posterior part of hindbody. *Scale-bars*: 200 µm.

**Additional file 4: Figure S3.** Photomicrographs of a paragenophore specimen of *Neocladocystis* sp., ventral view. *Scale-bars*: 200 µm.

**Additional file 5: Figure S4.** Photomicrographs of a paragenophore specimen of *Tanganyikatrema fusiforma* n. sp. **a** Body, ventral view. **b** Anterior body extremity with oral sucker. **c** Mid-body. **d** Posterior part of hindbody. *Scale-bars*: 100 µm.

**Additional file 6: Figure S5.** Photomicrographs of a paragenophore specimen of *Tanganyikatrema* sp. ‛elongataʼ. **a** Ventral view of immature specimen. **b** Anterior body extremity with oral sucker. **c** Mid-body. **d** Posterior part of hindbody. *Scale-bars*: 100 µm.

**Additional file 7: Figure S6.** Photomicrographs of *Grandifundilamena novemtestes* n. sp. **a** Body, ventral view. **b** Ovarian and testicular region. **c**, **d** Anterior body part. *Scale-bars*: 500 µm.

## Data Availability

The type- and voucher material is deposited at the Helminthological Collection of the Natural History Museum, London, UK, under the accession numbers NHMUK.2019.11.18.1–13, and in the collection of the Research Group Zoology: Biodiversity and Toxicology at Hasselt University in Diepenbeek, Belgium under the accession numbers HU 756–778. DNA sequences generated as a part of this study were deposited to the GenBank database under the accession numbers MN705808-MN705812 (*28S* rRNA gene) and MN702809-MN702817 (*cox*1).

## Abbreviations

NHM: Natural History Museum, London, UK
HU: Hasselt University, Hasselt, Belgium
*cox*1: cytochrome *c* oxidase subunit 1 gene
*28S* rDNA: large ribosomal subunit DNA
