# Supplementary material for: Uncharted digenean diversity in Lake Tanganyika: cryptogonimids (Digenea: Cryptogonimidae) infecting endemic lates perches (Actinopterygii: Latidae)
Source: Parasit Vectors. 2020 May 1;13:221. doi: 10.1186/s13071-020-3913-x (PMC7195733; doi:10.1186/s13071-020-3913-x)
Supplement: Supplementary file 1 — Additional file 1: Table S1. Summary data for 28S rDNA sequences retrieved from the GenBank database for species used in the phylogenetic analyses. [file 13071_2020_3913_MOESM1_ESM.docx]

**Additional file 1: Table S1.** Summary data for *28S* rDNA sequences retrieved from the GenBank database for species used in the phylogenetic analyses

| Digenean species | Host species | Locality, country | GenBank ID |  | Reference |
| --- | --- | --- | --- | --- | --- |
| *Acanthostomum burminis* (Bhalerao; 1926) | *Xenochrophis piscator* (Schneider) | Kanchanaburi Province, Thailand | KC489791 |  | [1] |
| *A. burminis* | “snail“ | Tiruchendur Beach, India | KM226898 |  | [2] |
| *A.* cf. *americanum* AMA-2017 | *Cichlasoma urophthalmum* (Günther) | Ria Celestun Biosphere Reserve, Yucatan Peninsula, Mexico | MG383499 |  | [3] |
| *Acanthostomum loossi* (Vigueras, 1957) | *C. urophthalmum* | Ria Celestun Biosphere Reserve, Yucatan Peninsula, Mexico | MG383502 |  | [3] |
| *Acanthostomum* sp. VVT-2013 | *Thiara scabra* (Müller) | Peradeniya, Sri Lanka | KC489792 |  | [1] |
| *Adlardia novaecaledoniae* Miller, Bray, Goiran, Justine & Cribb, 2009 | *Nemipterus furcosus* (Valenciennes) | Baie Maa, New Caledonia | FJ788496 |  | [4] |
| *Amphimerus ovalis* Barker, 1911 | *Trionyx muticus* (Lesueur) | Mississippi, USA | AY116876 |  | [5] |
| *Beluesca littlewoodi* Miller & Cribb, 2007 | *Plectorhinchus gibbosus* (Lacepède) | Lizard Island, Great Barrier Reef, Australia | EF566867 |  | [6] |
| *B. longicolla* Miller & Cribb, 2007 | *P. gibbosus* | Heron Island, Great Barrier Reef, Australia | EF566868 |  | [6] |
| *Caecincola parvulus* Marshall & Gilbert, 1905 | *Micropterus salmoides* (Lacepède) | USA | AY222231 |  | [5] |
| *Caulanus thomasi* Miller & Cribb, 2007 | *Lutjanus bohar* (Forsskål) | Lizard Island & Heron Island, Great Barrier Reef, Australia | EF428144 |  | [7] |
| *Centrocestus formosanus* (Nishigori, 1924) Price, 1932 | *Mesocricetus auratus* Waterhouse | Thailand | HQ874609 |  | [8] |
| *Chelediadema marjoriae* Miller & Cribb, 2007 | *Diagramma labiosum* Macleay | Lizard Island & Heron Island, Great Barrier Reef, Australia | EF566866 |  | [7] |
| *Cryptocotyle lingua* (Creplin, 1825) Fischoeder, 1903 | *Littorina littorea* (L.) | Isle of Sylt, North Sea, Germany | AY222228 |  | [5] |
| Cryptogonimidae gen. sp. SG-2019 | *Paralichthys patagonicus* Jordan | San Matias Gulf, Argentina | MK359083 |  | [9] |
| *Euryakaina manilensis* (Velasquez, 1961) Miller, Adlard, Bray, Justine & Cribb, 2010 | *Lutjanus vitta* (Quoy & Gaimard); *L. quinquelineatus* (Bloch) | Off Luzon Island, Philippines | HM056035 |  | [10] |
| *Eurycaina marinum* (Hafeezullah & Siddiqi) 1970 Miller, Adlard, Bray, Justine & Cribb, 2010 | *Lutjanus carponotatus* (Richardson); *L. fulviflamma* (Forsskål); *L. monostigma* (Cuvier)*; L. russellii* (Bleeker) | Off Tuticorin, India | HM056037 |  | [10] |
| *Euryakaina* sp. | *Lutjanus kasmira* (Forsskål) | Off Rasdhoo Atoll, Maldives | HM056036 |  | [10] |
| *Gynichthys diakidnus* Miller & Cribb, 2009 | *Plectorhinchus gibbosus* | Lizard Island & Heron Island, Great Barrier Reef, Australia | FJ907333 |  | [11] |
| *Latuterus maldivensis* Miller & Cribb, 2007 | *L. bohar* | Rasdhoo Atoll, Lizard Island, Great Barrier Reef, Australia | EF428146 |  | [7] |
| *L. tkachi* Miller & Cribb, 2007 | *L. bohar* | Lizard Island, Great Barrier Reef, Australia | EF428145 |  | [7] |
| *Lobosorchis polygongylus* Miller, Downie & Cribb, 2009 | *Nemipterus furcosus* (Valenciennes) | Lizard Island, Great Barrier Reef, Australia | FJ154902 |  | [12] |
| *L. tibaldiae* Miller & Cribb, 2005 | *L. fulviflamma* | Heron Island, Great Barrier Reef, Australia | FJ154901 |  | [12] |
| *Metadena lutiani* (Yamaguti, 1942) Miller & Cribb, 2008 | *L. bohar* | off the Great Barrier Reef, Australia | KF417630 |  | [4] |
| *Mitotrema anthostomatum* Manter, 1963 | *Cromileptes altivelis* (Valenciennes) | Australia | AY222229 |  | [5] |
| *Tanganyikatrema fusiforma* n. sp. | *Lates microlepis* Boulenger*,* | Mutondwe Island; Katukula; Mpulungu, Lake Tanganyika, Zambia | MN705811 |  | Present study |
| *Tanganyikatrema* sp. 'elongata' | *L. angustifrons, L. microlepis* | Mpulungu, Lake Tanganyika, Zambia | MN705812 |  | Present study |
| *Neocladocystis bemba* n. sp. Georgieva, Kmentová & Bray | *L. microlepis*, *L. angustifrons* | Mutondwe Island; Katukula; Mpulungu, Lake Tanganyika, Zambia | MN705808 |  | Present study |
| *N. biliaris* n. sp. Georgieva, Kmentová & Bray | *L. mariae* Steindachner | Uvira, Lake Tanganyika, DRC | MN705809 |  | Present study |
| *Neocladocystis* sp. | *L. angustifrons* | Mpulungu, Lake Tanganyika, Zambia | MN705810 |  | Present study |
| *Neometadena paucispina* Miller, Cutmore & Cribb, 2018 | *L. fulviflamma, L. russellii* | Off North Stradbroke Island, Moreton Bay, Australia | MH048926 |  | [13] |
| *Neometadena ovata* (Yamaguti, 1952) Miller & Cribb, 2008 | *L. carponotatus* | Off Lizard Island, Great Barrier Reef, Australia | EF116616 |  | [14] |
| *Retrovarium amplorificium* Miller & Cribb, 2007 | *Symphorus nematophorus* (Bleeker, 1860) | Lizard Island & Heron Island, Great Barrier Reef, Australia | EF116609 |  | [14] |
| *R. brooksi* Miller & Cribb, 2007 | *L. bohar, L. fulviflamma, L. gibbus* (Forsskål) | Heron Island, Rasdhoo Atoll, Moorea, Great Barrier Reef, Australia | EF116605 |  | [14] |
| *R. exiguiformosum* Miller & Cribb, 2007 | *S. nematophorus* | Lizard Island & Heron Island, Great Barrier Reef, Australia | EF116612 |  | [14] |
| *R. formosum* Miller & Cribb, 2007 | *S. nematophorus* | Lizard Island, Great Barrier Reef, Australia | EF116611 |  | [14] |
| *R. gardneri* Miller & Cribb, 2007 | *Lutjanus sebae* (Cuvier) | Heron Island, Great Barrier Reef, Australia | EF116606 |  | [14] |
| *R. manteri* Miller & Cribb, 2007 | *Lutjanus argentimaculatus* (Forsskål) | Lizard Island & Heron Island, Great Barrier Reef, Australia | EF116604 |  | [14] |
| *R. mariae* Miller & Cribb, 2007 | *D. labiosum* | Heron Island, Great Barrier Reef, Australia | EF116607 |  | [14] |
| *R. planum* Miller & Cribb, 2007 | *S. nematophorus* | Lizard Island & Heron Island, Great Barrier Reef, Australia | EF116614 |  | [14] |
| *R. sablae* Miller & Cribb, 2007 | *Aprion virescens* Valenciennes | Heron Island, Rasdhoo Atoll, Moorea, Great Barrier Reef, Australia | EF116608 |  | [14] |
| *R. snyderi* Miller & Cribb, 2007 | *S. nematophorus* | Lizard Island & Heron Island, Great Barrier Reef, Australia | EF116610 |  | [14] |
| *R. valdeparvum* Miller & Cribb, 2007 | *S. nematophorus* | Lizard Island & Heron Island, Great Barrier Reef, Australia | EF116613 |  | [14] |
| *Siphodera vinaledwardsii* (Linton, 1901) Linton, 1910 | *Sciaenops ocellatus* (L.) | Gulf of Mexico, South of Horn Island, Mississippi, USA | AY222230 |  | [5] |
| *Siphoderina grunnitus* Miller & Cribb, 2008 | *Plectorhinchus gibbosus* (Lacepède) | Lizard Island, Great Barrier Reef, Australia | EU571261 |  | [15] |
| *S. hirastricta* (Manter, 1963) Miller & Cribb, 2008 | *L. argentimaculatus* | Off Lizard Island, Great Barrier Reef, Queensland, Australia; Ningaloo Reef, Western Australia; Rasdhoo Atoll, Maldives. | EU571260 |  | [15] |
| *S. infirma* Miller & Cribb, 2008 | *L. russelli* | Lizard Island, Great Barrier Reef, Australia | EU571264 |  | [15] |
| *S. jactus* Miller & Cribb, 2008 | *L. fulviflamma* | Heron Island, Great Barrier Reef, Australia | EU571263 |  | [15] |
| *S. poulini* Miller & Cribb, 2008 | *L. argentimaculatus* | North Stradbroke Island, Moreton Bay, Queensland, Australia | EU571267 |  | [15] |
| *S. quasispina* Miller & Cribb, 2008 | *L. fulviflamma* | Heron Island, Great Barrier Reef, Australia | EU571265 |  | [15] |
| *S. subuterus* Miller & Cribb, 2008 | *Lutjanus adetii* (Castelnau) | Heron Island, Great Barrier Reef, Australia | EU571266 |  | [15] |
| *S. territans* Miller & Cribb, 2008 | *Lutjanus carponotatus* (Richardson) | Heron Island, Great Barrier Reef, Australia | EF116615 |  | [15] |
| *S. virga* Miller & Cribb, 2008 | *L. russelli* | North Stradbroke Island, Moreton Bay, Queensland, Australia | EU571262 |  | [15] |
| *Siphomutabilis gurukun* (Machida, 1986) Miller & Cribb, 2013 | *Caesio cuning* (Bloch); *Caesio caerulaurea* (Lacepède) | Off Lizard Island, Great Barrier Reef, Queensland, Australia; Ningaloo Reef, Western Australia; Rasdhoo Atoll, Maldives | KF417631 |  | [4] |
| *S. raritas* Miller & Cribb, 2013 | *C. cuning* | Off Lizard Island, Great Barrier Reef, Queensland, Australia; Ningaloo Reef, Western Australia; Rasdhoo Atoll, Maldives | KF417632 |  | [4] |
| *Varialvus charadrus* Miller, Bray, Justine & Cribb, 2010 | *L. vitta, L. bohar, L. carponotatus, L. fulviflamma, L. fulvus, L. gibbus, L. kasmira, L. quinquelineatus* | Lizard Island, Great Barrier Reef, Australia | HM187778 |  | [16] |
| *V. jenae* Miller, Bray, Justine & Cribb, 2010 | *L. carponotatus* | Lizard Island, Great Barrier Reef, Australia | HM187776 |  | [16] |
| *V. lacertus* Miller, Bray, Justine & Cribb, 2010 | *L. quinquelineatus; L. fulvus* | Lizard Island, Great Barrier Reef, Australia | HM187777 |  | [16] |

**References**

1. Jayawardena UA, Tkach V V., Navaratne AN, Amerasinghe PH, Rajakaruna RS. Malformations and mortality in the Asian Common Toad induced by exposure to pleurolophocercous cercariae (Trematoda: Cryptogonimidae). Parasitol Int. 2013;62:246**–**52.

2. Arya LK, Rathinam SR, Lalitha P, Kim UR, Ghatani S, Tandon V. Trematode fluke *Procerovum varium* as cause of ocular inflammation in children, south India. Emerg Infect Dis. 2016;22:192**–**200.

3. Martínez-Aquino A, Vidal-Martínez VM, Aguirre-Macedo ML. A molecular phylogenetic appraisal of the acanthostomines *Acanthostomum* and *Timoniella* and their position within Cryptogonimidae (Trematoda: Opisthorchioidea). PeerJ. 2017;5:e4158.

4. Miller TL, Cribb TH. Dramatic phenotypic plasticity within species of *Siphomutabilus* n. g. (Digenea: Cryptogonimidae) from Indo-Pacific caesionines (Perciformes: Lutjanidae). Syst Parasitol. 2013;86:101**–**112.

5. Olson PD, Cribb TH, Tkach VV, Bray RA, Littlewood DTJ. Phylogeny and classification of the Digenea (Platyhelminthes: Trematoda). Int J Parasitol. 2003;33:733**–**55.

6. Miller T, Cribb T. Two new cryptogonimid genera *Beluesca* n. gen. and *Chelediadema* n. gen. (Digenea: Cryptogonimidae) from tropical Indo-West Pacific Haemulidae (Perciformes). Zootaxa. 2007;1543:45**–**60.

7. Miller T, Cribb T. Two new cryptogonimid genera (Digenea, Cryptogonimidae) from *Lutjanus bohar* (Perciformes, Lutjanidae): analyses of ribosomal DNA reveals wide geographic distribution and presence of cryptic species. Acta Parasitol. 2007;52:104**–**113.

8. Wongsawad C, Wongsawad P, Sukontason K, Maneepitaksanti W, Nantarat N. Molecular phylogenetics of *Centrocestus formosanus* (Digenea: Heterophyidae) originated from freshwater fish from Chiang Mai Province, Thailand. Korean J Parasitol. 2017;55:31**–**7.

9. Hernández-Orts JS, Georgieva S, Landete DN, Scholz T. Heterophyid trematodes (Digenea) from penguins: A new species of *Ascocotyle* Looss, 1899, first description of metacercaria of *Ascocotyle (A.) patagoniensis* Hernández-Orts et al. (2012), and first molecular data. Int J Parasitol Parasites Wildl. 2019;8:94**–**105.

10. Miller TL, Adlard RD, Bray RA, Justine J-. L, Cribb TH. Cryptic species of *Euryakaina* n. g. (Digenea: Cryptogonimidae) from sympatric lutjanids in the Indo-West Pacific. Syst Parasitol. 2010;77:185**–**204.

11. Miller TL, Cribb TH. *Gynichthys diakidnus* n. g., n. sp. (Digenea: Cryptogonimidae) from the grunt *Plectorhinchus gibbosus* (Lacépède, 1802) (Perciformes: Haemulidae) off the Great Barrier Reef, Australia. Syst Parasitol. 2009;74:103**–**112.

12. Miller TL, Downie AJ, Cribb TH. Morphological disparity despite genetic similarity; new species of *Lobosorchis* Miller & Cribb, 2005 (Digenea: Cryptogonimidae) from the Great Barrier Reef and the Maldives. Zootaxa. 2009;1992:37**–**52.

13. Miller TL, Cutmore SC, Cribb TH. Two species of *Neometadena* Hafeezullah & Siddiqi, 1970 (Digenea: Cryptogonimidae) from Moreton Bay, Australia, including the description of *Neometadena paucispina* n. sp. from Australian Lutjanidae. Syst Parasitol. 2018;95:655**–**64.

14. Miller TL, Cribb TH. Coevolution of *Retrovarium* n. gen. (Digenea: Cryptogonimidae) in Lutjanidae and Haemulidae (Perciformes) in the Indo-West Pacific. Int J Parasitol. 2007;37:1023**–**45.

15. Miller T, Cribb T. Eight new species of *Siphoderina* Manter, 1934 (Digenea, Cryptogonimidae) infecting Lutjanidae and Haemulidae (Perciformes) off Australia. Acta Parasitol. 2008;53:344**–**64.

16. Miller T, Bray R, Justine J-L, Cribb T. *Varialvus* gen. nov. (Digenea, Cryptogonimidae), from species of Lutjanidae (Perciformes) off the Great Barrier Reef, New Caledonia and the Maldives. Acta Parasitol. 2010;55:327**–**39.
