# Supplementary figures and images for: Uncharted digenean diversity in Lake Tanganyika: cryptogonimids (Digenea: Cryptogonimidae) infecting endemic lates perches (Actinopterygii: Latidae)
Source: Parasit Vectors. 2020 May 1;13:221. doi: 10.1186/s13071-020-3913-x (PMC7195733; doi:10.1186/s13071-020-3913-x)

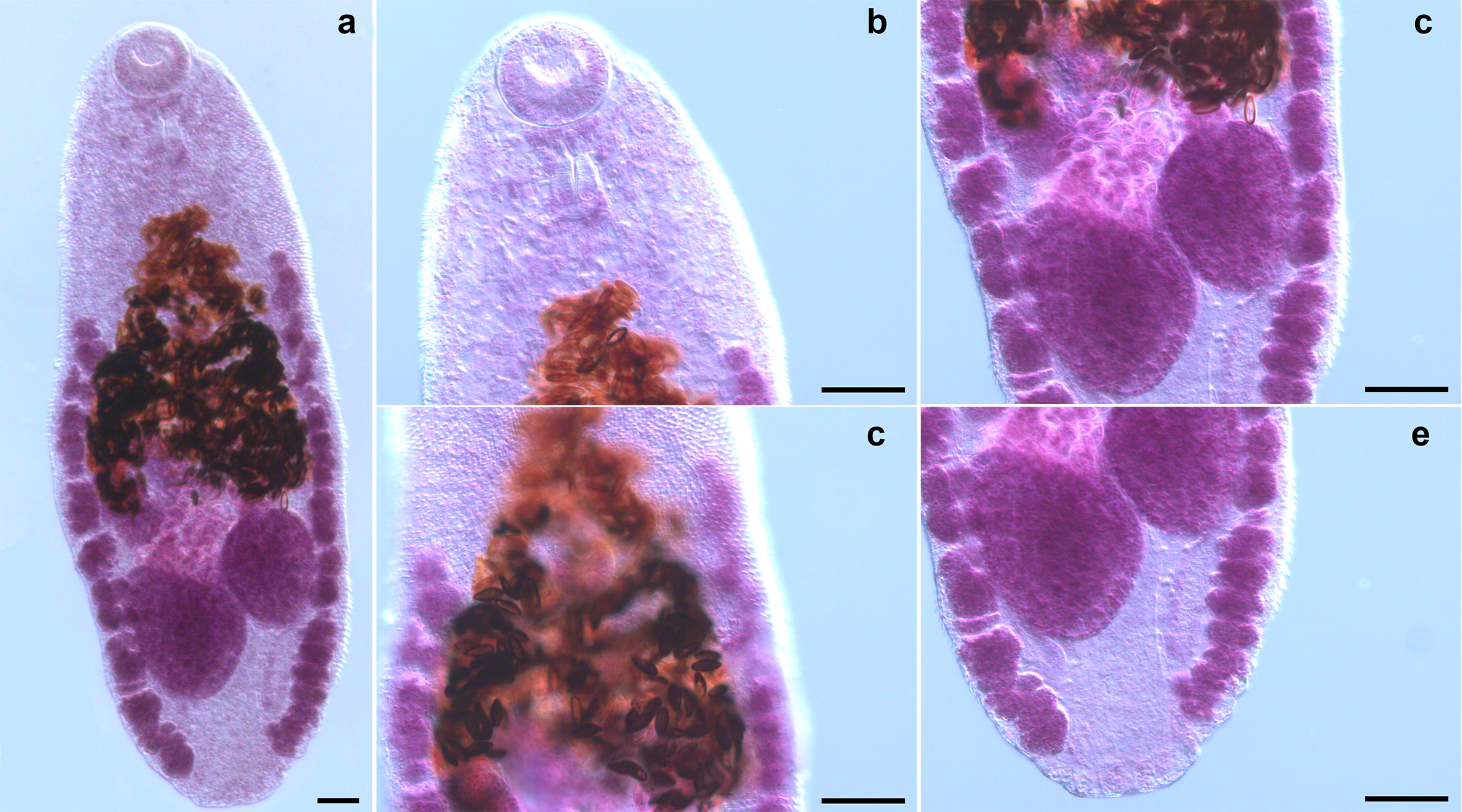

Supplement: Supplementary file 2 — Additional file 2: Figure S1. Photomicrographs of a paragenophore specimen of Neocladocystis bemba n. sp. a Body, ventral view. b Anterior body extremity with oral sucker. c Mid-body. d Ovarian and testicular region. e Posterior part of hindbody. Scale-bars: 100 µm. [file 13071_2020_3913_MOESM2_ESM.tif]

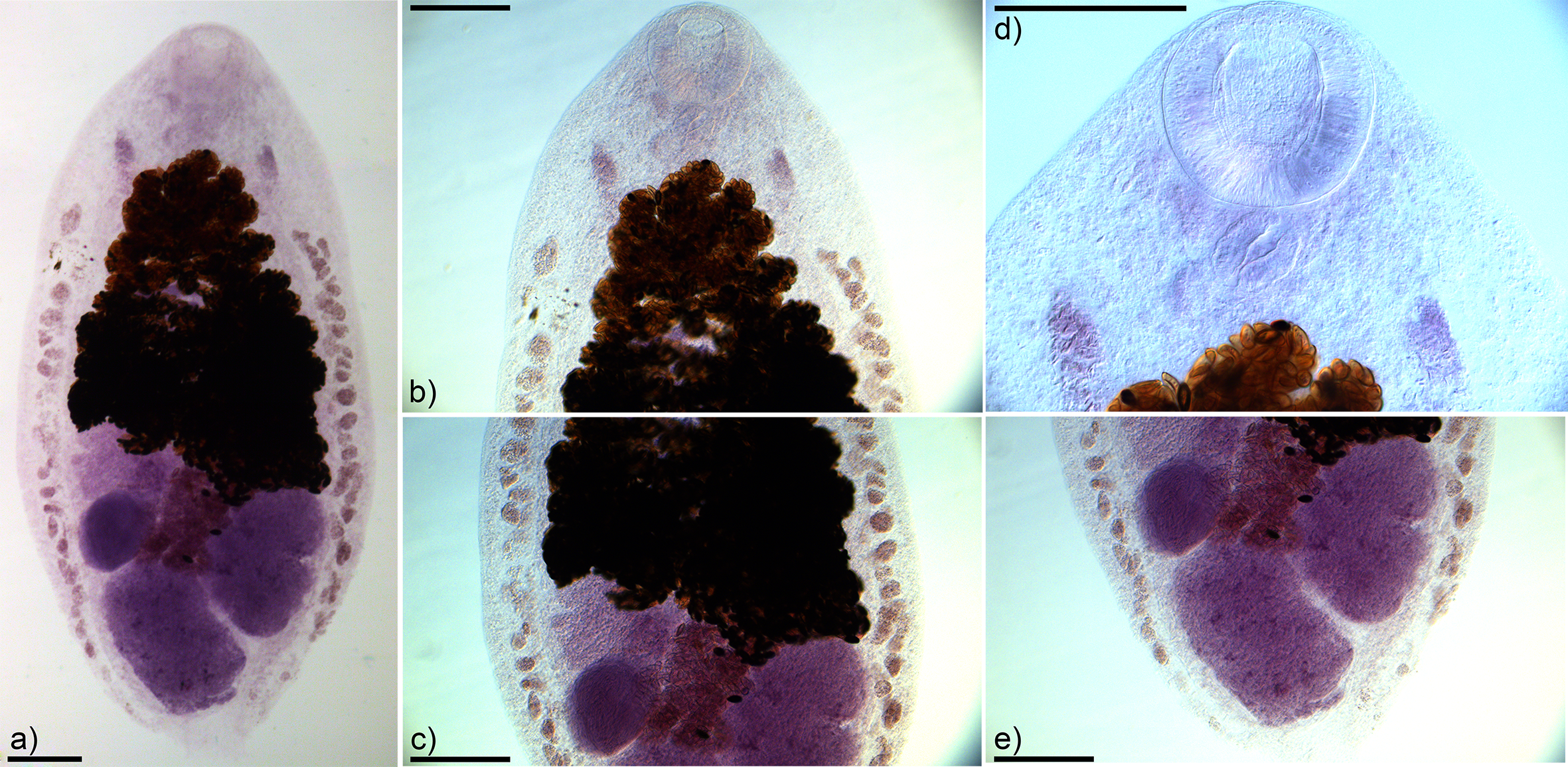

Supplement: Supplementary file 3 — Additional file 3: Figure S2. Photomicrographs of a paragenophore specimen of Neocladocystis biliaris n. sp. a Body, ventral view. b Anterior body extremity with oral sucker. c Mid-body. d Ovarian and testicular region. e Posterior part of hindbody. Scale-bars: 200 µm. [file 13071_2020_3913_MOESM3_ESM.tif]

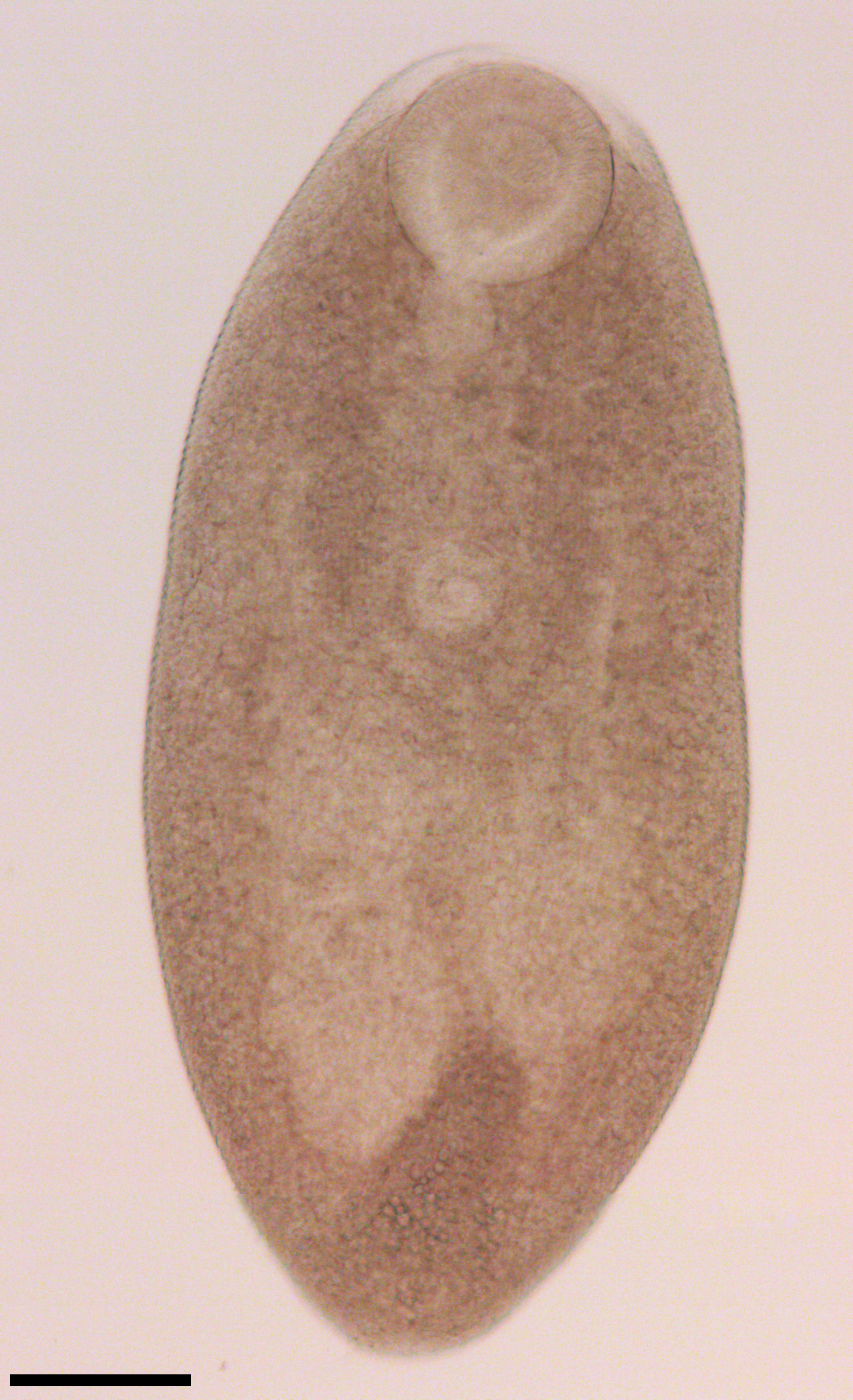

Supplement: Supplementary file 4 — Additional file 4: Figure S3. Photomicrographs of a paragenophore specimen of Neocladocystis sp., ventral view. Scale-bars: 200 µm. [file 13071_2020_3913_MOESM4_ESM.tif]

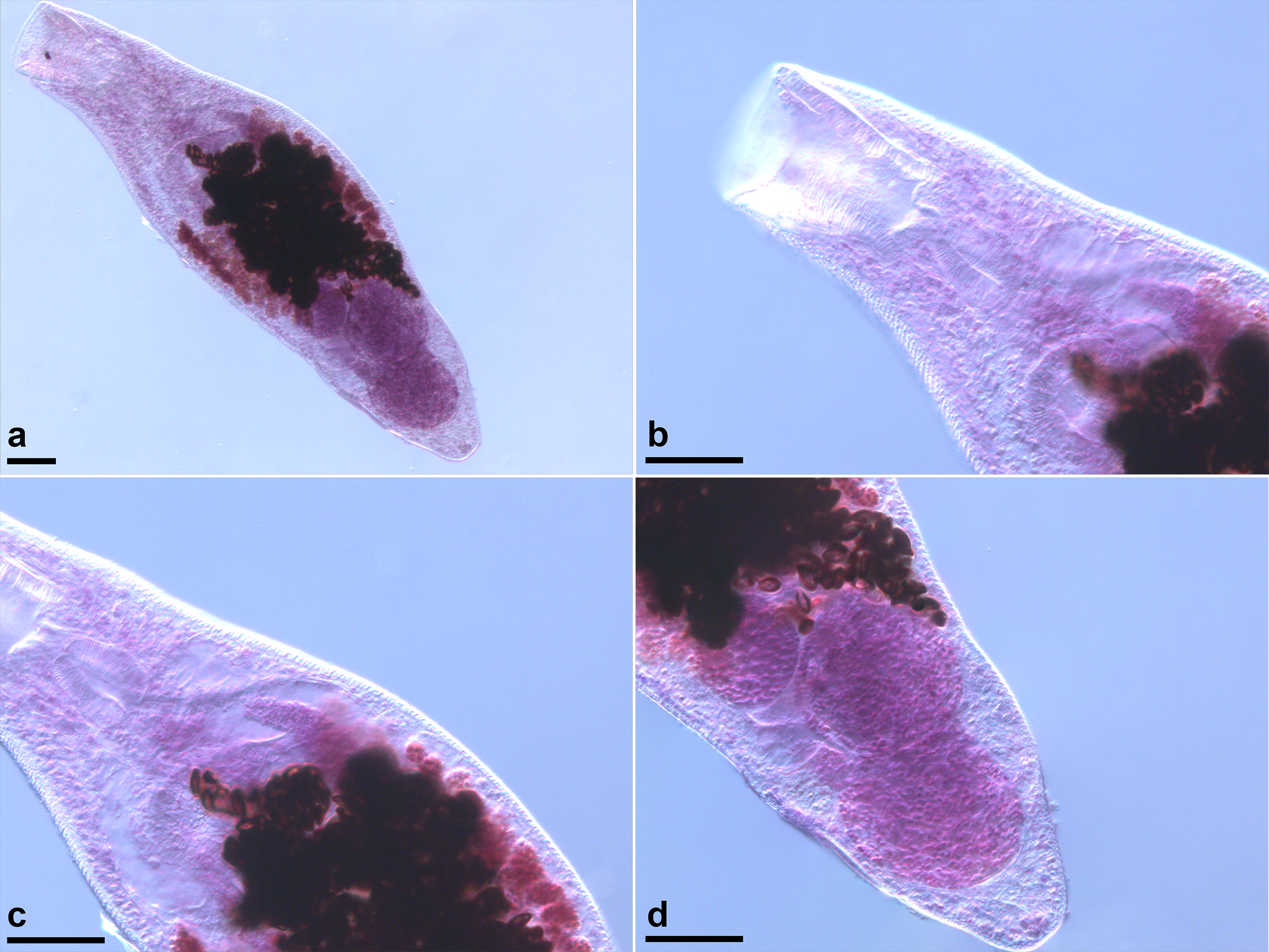

Supplement: Supplementary file 5 — Additional file 5: Figure S4. Photomicrographs of a paragenophore specimen of Tanganyikatrema fusiforma n. sp. a Body, ventral view. b Anterior body extremity with oral sucker. c Mid-body. d Posterior part of hindbody. Scale-bars: 100 µm. [file 13071_2020_3913_MOESM5_ESM.tif]

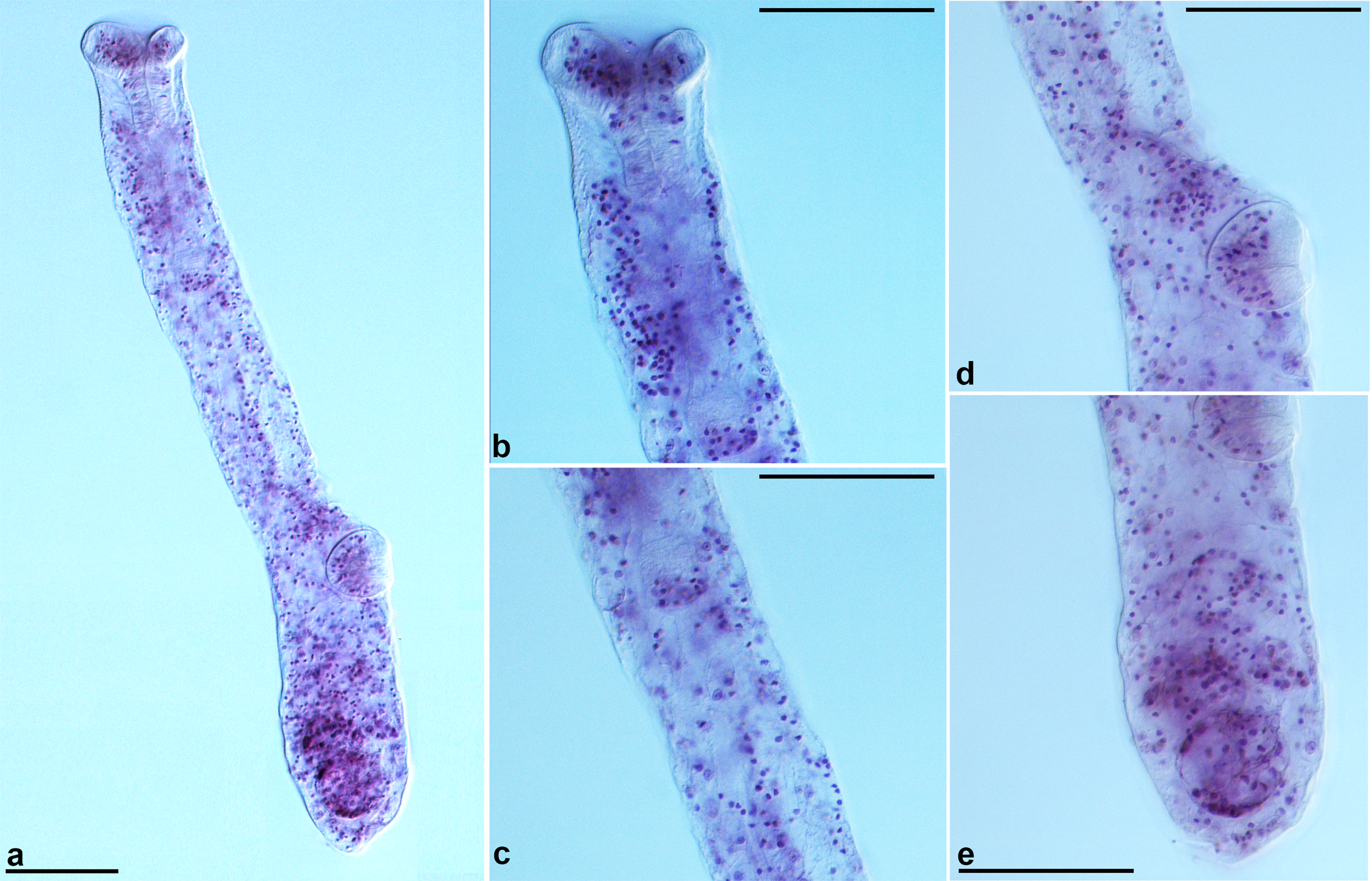

Supplement: Supplementary file 6 — Additional file 6: Figure S5. Photomicrographs of a paragenophore specimen of Tanganyikatrema sp. ‛elongataʼ. a Ventral view of immature specimen. b Anterior body extremity with oral sucker. c Mid-body. d Posterior part of hindbody. Scale-bars: 100 µm. [file 13071_2020_3913_MOESM6_ESM.tif]

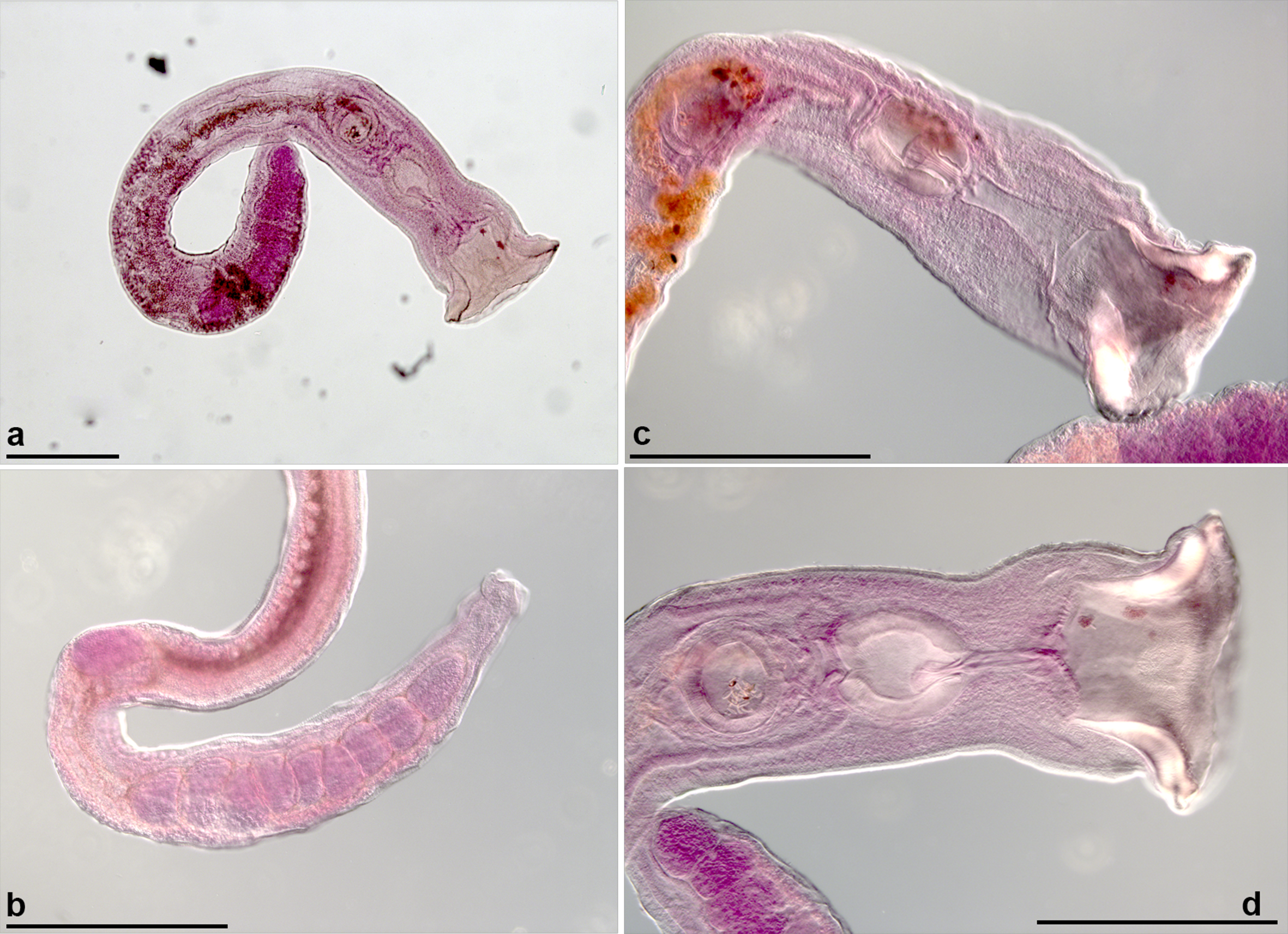

Supplement: Supplementary file 7 — Additional file 7: Figure S6. Photomicrographs of Grandifundilamena novemtestes n. sp. a Body, ventral view. b Ovarian and testicular region. c, d Anterior body part. Scale-bars: 500 µm. [file 13071_2020_3913_MOESM7_ESM.tif]
